# Potential role of lipophagy impairment for anticancer effects of glycolysis-suppressed pancreatic ductal adenocarcinoma cells

**DOI:** 10.1038/s41420-024-01933-4

**Published:** 2024-04-05

**Authors:** Zhiheng Zhang, Haruna Aoki, Keitaro Umezawa, Joshua Kranrod, Natsumi Miyazaki, Taichi Oshima, Takuya Hirao, Yuri Miura, John Seubert, Kousei Ito, Shigeki Aoki

**Affiliations:** 1 Laboratory of Biopharmaceutics, Graduate School of Pharmaceutical Sciences, Chiba University, 1-8-1 Inohana, Chuo-ku, Chiba-city, Chiba, 260-8675 Japan; 2 Research Team for Mechanism of Aging, Tokyo Metropolitan Institute of Gerontology, 35‑2 Sakae‑cho, Itabashi‑ku, Tokyo, 173‑0015 Japan; 3 Faculty of Pharmacy and Pharmaceutical Sciences, University of Alberta, 2026-M Katz Group Centre for Pharmacy and Health Research, 11361-97 Ave, Edmonton, AB T6G 2E1 Canada; 4 Divisions of Clinical Pharmacokinetics, Department of Pharmaceutical Sciences, International University of Health and Welfare, 2600-1 Kitakanemaru, Ohtawara, Tochigi 324‐8501 Japan; 5 Department of Pharmacology, Faculty of Medicine and Dentistry, University of Alberta, Edmonton, AB T6G 2E1 Canada

**Keywords:** Cancer metabolism, Target identification

## Abstract

Although increased aerobic glycolysis is common in various cancers, pancreatic ductal adenocarcinoma (PDAC) cells can survive a state of glycolysis suppression. We aimed to identify potential therapeutic targets in glycolysis-suppressed PDAC cells. By screening anticancer metabolic compounds, we identified SP-2509, an inhibitor of lysine-specific histone demethylase 1A (LSD1), which dramatically decreased the growth of PDAC PANC-1 cells and showed an anti-tumoral effect in tumor-bearing mice. The growth of glycolysis-suppressed PANC-1 cells was also inhibited by another LSD1 inhibitor, OG-L002. Similarly, the other two PDAC cells (PK-1 and KLM-1) with suppressed glycolysis exhibited anticancer effects against SP-2509. However, the anticancer effects on PDAC cells were unrelated to LSD1. To investigate how PDAC cells survive in a glycolysis-suppressed condition, we conducted proteomic analyses. These results combined with our previous findings suggested that glucose-starvation causes PDAC cells to enhance mitochondrial oxidative phosphorylation. In particular, mitochondrial fatty acid metabolism was identified as a key factor contributing to the survival of PDAC cells under glycolysis suppression. We further demonstrated that SP-2509 and OG-L002 disturbed fatty acid metabolism and induced lipid droplet accumulation through the impairment of lipophagy, but not bulk autophagy. These findings indicate a significant potential association of lipophagy and anticancer effects in glycolysis-suppressed PDAC cells, offering ideas for new therapeutic strategies for PDAC by dual inhibition of glycolysis and fatty acids metabolism.

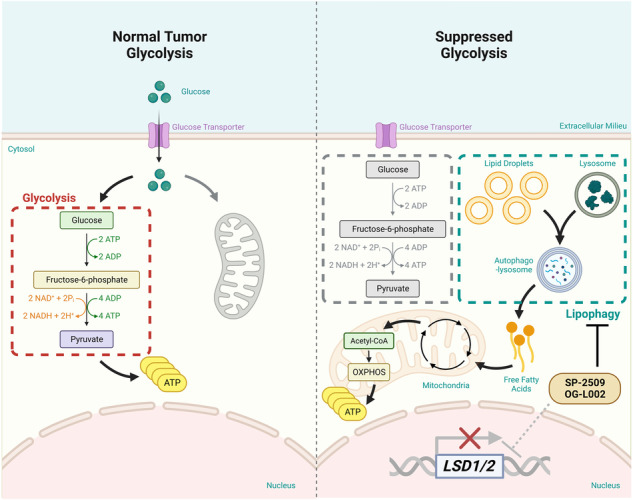

## Introduction

Glycolysis is generally less efficient in producing ATP than mitochondrial oxidative phosphorylation (OXPHOS). However, tumors often display high levels of glucose uptake and lactate production via aerobic glycolysis, even with adequate oxygen available, a phenomenon known as the Warburg effect [1]. The molecular mechanisms by which cancer cells upregulate glycolysis have attracted substantial research attention. Among the proposed mechanisms, the AKT oncogene has been shown to enhance glycolytic flux by activating glucose transporters without affecting mitochondrial OXPHOS, thereby maintaining high levels of ATP [2]. Hypoxia-inducible factor 1α also promotes a preferential dependence on glycolysis by inducing the transcription of glycolytic enzymes such as hexokinase (HK) and lactate dehydrogenase A (LDHA) [3]. Activation of pyruvate dehydrogenase (PDH) kinase-1 (PDK-1) inactivates PDH, preventing the entry of pyruvate into the mitochondrial tricarboxylic acid (TCA) cycle [4]. In contrast, inhibition of HK2, which catalyzes the rate-limiting and first obligatory step of glucose metabolism, can markedly reduce the proliferation of lung cancer cells [5]. Therefore, the glycolytic pathway, which includes glucose transporters and glycolytic enzymes, has been targeted for cancer therapy.

Some cancer cells survive and even continue aggressive proliferation when glucose is depleted [6]. For example, breast cancer cells can survive a state of glycolysis suppression by modulating mitochondrial metabolism, including OXPHOS, and instead favoring glutamine utilization during glucose starvation [6]. Cancer cells can compensate for glucose depletion by activating various metabolic pathways, such as fatty acid (FA) oxidation [7]. For example, breast cancer cells ubiquitously express carnitine palmitoyltransferase (CPT) 1C, which promotes energy production through increased FA oxidation, conferring the cells with resistance to glucose starvation [8]. FA metabolism includes complex molecular processes, including FA uptake and the de novo synthesis of FAs. Colorectal and pancreatic cancer cells may favor de novo synthesis rather than FAs uptake from the tumor microenvironment, as they highly express the gene encoding FA synthase [9]. These findings suggest that cancer cells can proliferate under conditions of glycolysis suppression if they can compensate by utilizing other adaptive processes such as FA oxidation.

Several metabolic pathways that are common in pancreatic cancer may be lethal to the cancer cells during their early stages of development, providing an opportunity to identify novel therapeutic targets [10]. Approximately half of all patients with resected pancreatic ductal adenocarcinomas (PDAC) have a poor prognosis, and cancer recurrence and metastasis may occur even after successful surgery [10]. Patients with pancreatic cancer may also show resistance to chemotherapy such as gemcitabine [10]. Therefore, further research is required to identify the molecular vulnerabilities of pancreatic cancer. Pancreatic adenocarcinoma is a major form of pancreatic cancer, with PDAC being the most prevalent subtype [11]. PDAC arises from three precursors: pancreatic intraepithelial neoplasia, intraductal papillary mucinous neoplasms, and mucinous cystic neoplasms [11]. Over 90% of patients with PDAC harbor activating mutations in the KRAS oncogene, suggesting that KRAS may be a critical driver of tumorigenesis. The KRAS-G12C mutation is common in non-small cell lung cancer, and specific inhibitors of this mutation, such as sotorasib, have shown remarkable anticancer efficacy. However, the G12C mutation is rare in PDAC and there is currently no effective pharmacological inhibitor targeting the more common KRAS G12D/V mutation associated with PDAC [12]. Therefore, the aim of this study was to identify the metabolic pathways of PDAC that can serve as new therapeutic targets.

KRAS specifically regulates rate-limiting glycolytic enzymes, and therefore the metabolic pathways activated downstream of KRAS have been widely studied as potential therapeutic targets. However, PDAC cells may be resistant to glycolysis inhibitors, such as LDHA inhibitors [13], suggesting compensatory mechanisms enabling PDAC cell survival in the face of glycolysis suppression. Our previous study showed that glucose starvation failed to lower the survival rate of PDAC cells, which reprogrammed their glucose metabolism mode to OXPHOS [14]. Therefore, in this study, we explored the in-depth compensatory metabolic mechanism occurring in PDAC cells when glycolysis is suppressed, which can help to identify specific therapeutic targets for improving the treatment of PDAC.

## Results

### PDAC cells with suppressed glycolysis exhibit anticancer effects against SP-2509

**Fig. 1:**
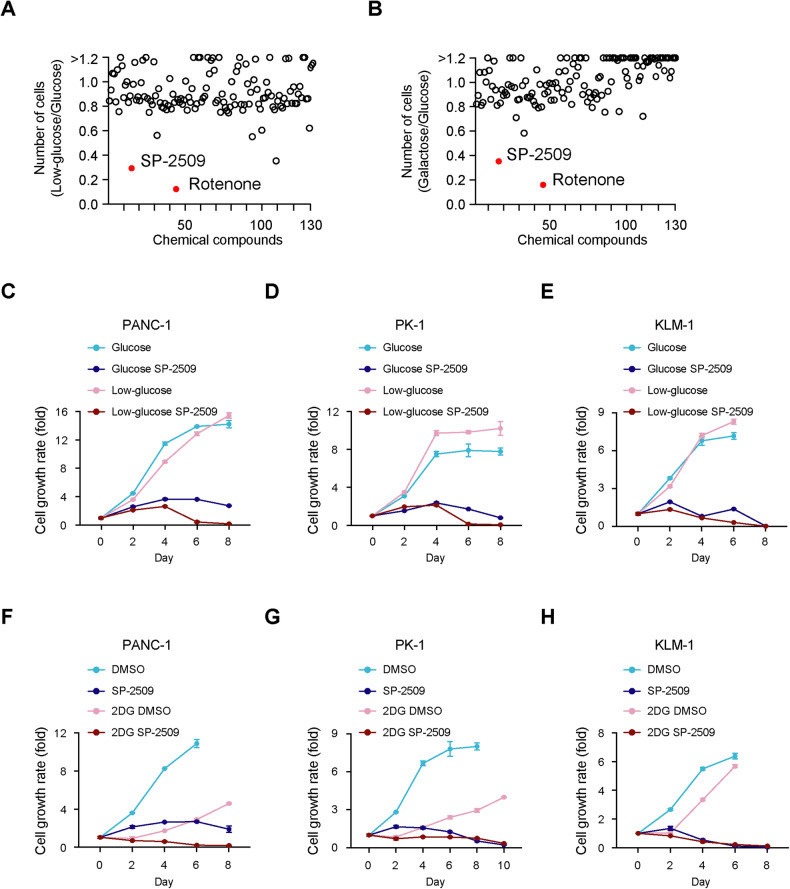
PDAC cells with suppressed glycolysis exhibit anticancer effects against SP-2509. **A**, **B** PANC-1 cells were cultured in glucose, low-glucose, or galactose medium with each compound (10 µM) for 48 h. The cell number was determined via an MTT assay. The number of cells treated with each compound in the presence of low-glucose/glucose or galactose/glucose are indicated in **A** and **B**, respectively. Raw data are shown in Supplementary Table 1. PANC-1 (**C**), PK-1 (**D**), and KLM-1 (**E**) cells were cultured in glucose or low-glucose medium with or without SP-2509 (10 μM) for eight days. The cell number was determined via an MTT assay. The level of produced formazan crystals was quantified each day. Data at each timepoint are normalized against day 0. Data are presented as mean ± SD for experiments performed in triplicate. PANC-1 (**F**), PK-1 (**G**), and KLM-1 (**H**) cells treated with or without 2-deoxy-d-glucose (2DG; 10 mM for PANC-1, 1 mM for PK-1 and KLM-1) were cultured with or without SP-2509 (10 µM) for eight or ten days. The cell number was determined via an MTT assay. The level of produced formazan crystals was quantified each day. Data at each timepoint are normalized against day 0. Data are presented as mean ± SD for experiments performed in triplicate.

PDAC PANC-1 cells were cultured in either a low-glucose or galactose culture medium to mimic glycolysis suppression [14]. To determine the potential therapeutic targets under these conditions, we screened a library of 130 compounds and identified that rotenone and SP-2509 dramatically decreased the number of cells cultured in the presence of both low-glucose levels and galactose (Fig. 1A, B and Supplementary Table 1).

Since our previous study indicated that glycolysis-suppressed PANC-1 cells were highly sensitive to rotenone, an inhibitor of mitochondrial electron transport chain (ETC) complex I [14], in this study, we focused on the potential inhibitory effects of SP-2509, which dramatically decreased the growth of all PDAC cells (PANC-1, PK-1, and KLM-1) under low-glucose conditions (Fig. 1C–E, Supplementary Fig. 1A–C).

Moreover, we found that PDAC cells undergo metabolic reprogramming to mitochondrial OXPHOS after exposure to 2-deoxy-d-glucose (2DG), an inhibitor of glycolysis that mimics glucose deprivation, which has been proposed as an antitumor agent [15] (Supplementary Fig. 2A–F). Treatment of SP-2509 in combination with 2DG dramatically decreased the growth of all PDAC cells (Fig. 1F–H). Taken together, the mechanisms underlying the response of glycolysis-suppressed PDAC cells to SP-2509 may hold therapeutic value.

### LSD1 has limited involvement in the anticancer response of PDAC cells against LSD1 inhibitors

**Fig. 2:**
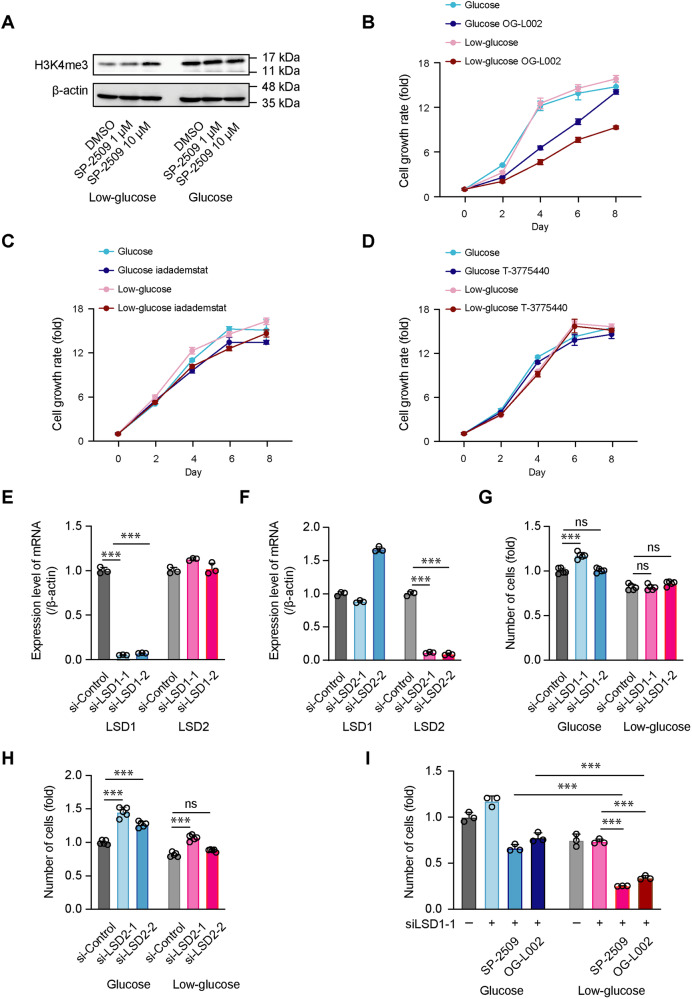
LSD1 has limited involvement in the anticancer response of PDAC cells against LSD1 inhibitors. **A** PANC-1 cells were cultured in low-glucose or glucose media with DMSO or SP-2509 (1 and 10 µM) for 48 h. The cell lysates were subjected to western blotting for tri-methyl-histone H3 (Lys4) (H3K4me3) and β-actin antibodies. PANC-1 cells were cultured in glucose or low-glucose medium with or without OG-L002 (50 µM) (**B**), iadademstat (100 µM) (**C**), or T-3775440 (50 µM) (**D**) for eight days. The cell number was determined via an MTT assay. The level of produced formazan crystals was quantified each day. Data at each timepoint are normalized against day 0. Data are presented as mean ± SD for experiments performed in triplicate. PANC-1 cells were transfected with si-control, si-lysine-specific demethylase 1 (LSD) 1 (**E**), or si-LSD2 (**F**) for 24 h and then the medium was replaced. Transfected cells were cultured with glucose medium for another 48 h. RT-qPCR analysis was performed to measure the mRNA levels of LSD1, LSD2, and β-actin genes. Data are normalized against the mRNA levels of LSD1 and LSD2 in cells transfected with si-control. Data are presented as mean ± SD for experiments performed in triplicate. Statistical analysis is based on two-way ANOVA followed by Tukey’s test for multiple comparisons. PANC-1 cells were transfected with si-control, si-LSD1 (**G**), or si-LSD2 (**H**) for 24 h and then the medium was replaced. Transfected cells were cultured with glucose or low-glucose medium for another 48 h. The number of cells was determined via an MTT assay. Data are normalized against the level of formazan crystals produced in si-control-transfected cells cultured in glucose. Data are presented as mean ± SD for experiments performed in quintuple. Statistical analysis is based on two-way ANOVA followed by Tukey’s test for multiple comparisons. **I** PANC-1 cells were transfected with si-control or si-LSD1-1 for 24 h and then the medium was replaced. Transfected cells were cultured in glucose or low-glucose medium with or without SP-2509 (10 µM) or OG-L002 (50 µM) for another 48 h. Data are presented as mean ± SD for experiments performed in triplicate. Statistical analysis is based on two-way ANOVA followed by Tukey’s test for multiple comparisons. ***P < 0.001; ns not significant.

SP-2509 is known as a potent and reversible inhibitor of lysine-specific histone demethylase 1 A (LSD1) [16], and we also confirmed that LSD1 expression was inhibited by treatment with SP-2509 under low-glucose conditions (Fig. 2A and Supplementary Fig. 3A). We further tested the effect of different concentrations of SP-2509 on PDAC cell growth. With treatment of 0.3 µM SP-2509, there remained a substantial number of PDAC cells, whereas at 10 µM, the growth of low glucose-cultured PDAC cells was dramatically decreased (Supplementary Fig. 1). Given that the half-maximal inhibitory concentration (IC50) of SP-2509 for LSD1 is only 13 nM, we tested the effects of various concentrations of other known LSD1 inhibitors (OG-L002, iadademstat, and T-3775440) to confirm the limited involvement of LSD1 in the observed anticancer effects of SP-2509 against PDAC cells. Suppression of cell growth was observed under low-glucose conditions when the concentration of OG-L002 was increased to 50 µM (the IC50 for LSD1 is 20 nM) (Fig. 2B and Supplementary Fig. 4A), while no suppression of cell growth was observed with treatment of iadademstat and T-3775440 with an IC50 for LSD1 of 20 nM and 2.1 nM, respectively (Fig. 2C, D, Supplementary Fig. 4B, C). Moreover, depletion of LSD1/2 did not decrease the number of low glucose-cultured PANC-1 cells (Fig. 2E–H and Supplementary Fig. 5), whereas treatment with both SP-2509 and OG-L002 significantly decreased the number of LSD1-depleted and low glucose-cultured PDAC cells (Fig. 2I). These results suggested that the anticancer effects observed in glycolysis-suppressed PDAC cells against these two LSD1 inhibitors are minimally influenced by LSD1. Therefore, we further employed SP-2509 and OG-L002 as tools to explore the underlying mechanism of inhibition and identify new therapeutic targets for PDAC cells.

### Quantitative proteomic analysis of PANC-1 cells in low-glucose conditions

**Fig. 3:**
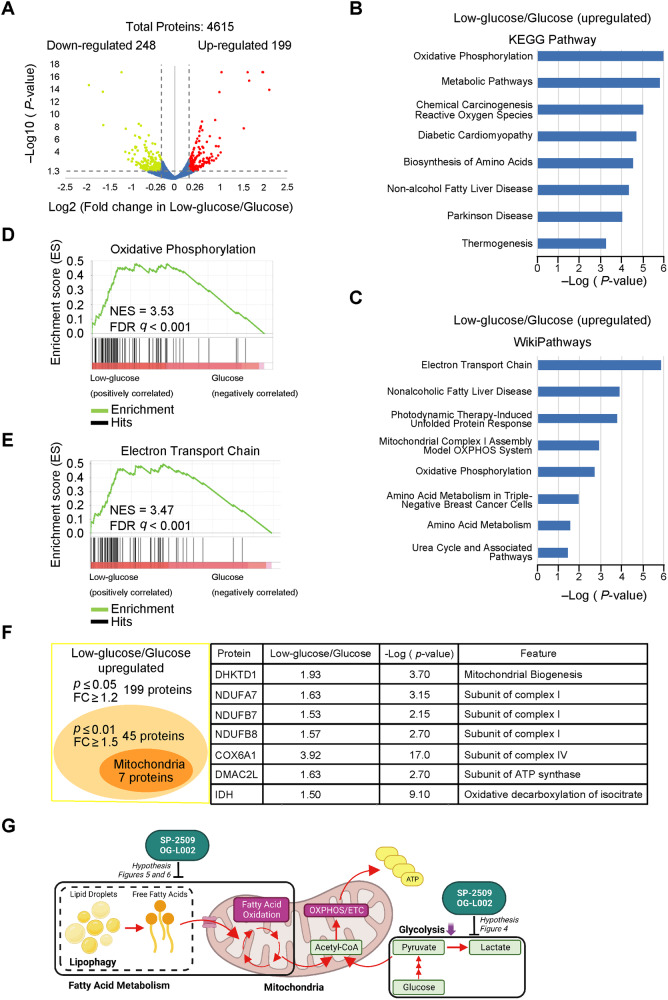
Quantitative proteomic analysis of PANC-1 cells cultured in low-glucose conditions. **A** PANC-1 cells were cultured in glucose or low-glucose medium for 6 days in triplicate. The volcano plot shows overall differentially expressed proteins of PANC-1 cells identified with a *P*-value ≤ 0.05 and low-glucose/glucose ≥1.2-fold cutoff criteria. Proteins with downregulated or upregulated expression are shown in green or red, respectively. **B**, **C** Kyoto Encyclopedia of Genes and Genomes (KEGG) pathway (**B**) and WikiPathways (**C**) analysis of proteins with upregulated expression in the low-glucose condition. The vertical axis represents the pathway and the horizontal axis represents the enrichment score [–log(*P*-value)]. Gene set enrichment analysis in the oxidative phosphorylation KEGG pathway (**D**) and electron transport chain of WikiPathways (**E**) showing enrichment of proteins whose levels increased in PANC-1 cells cultured in the low-glucose condition. NES, normalized enrichment score. FDR, false discovery rate. **F** Overlap between low-glucose/glucose-upregulated and related mitochondrial proteins. Details of mitochondrion-related proteins are shown in a list. FC fold change, DHKTD1 probable 2-oxoglutarate dehydrogenase e1 component, NDUFA7 NADH: ubiquinone oxidoreductase subunit a7, NDUFB7 NADH: ubiquinone oxidoreductase subunit b7, NDUFB8 NADH: ubiquinone oxidoreductase subunit b8, COX6A1 cytochrome c oxidase subunit 6a1, DMAC2L distal membrane arm assembly component 2 like, IDH isocitrate dehydrogenases. **G** Schematic of the hypothesis explaining the decrease of ATP levels by SP-2509 or OG-L002 in glycolysis-suppressed PDAC cells.

To clarify the influence of glycolytic suppression on PDAC cellular metabolism, the differentially expressed proteins of PANC-1 cells cultured under glucose and low-glucose conditions were identified by quantitative proteomic analysis. Among the total 4 615 proteins that were detected and quantified (Fig. 3A), 447 differentially expressed proteins were identified according to a *P*-value ≤ 0.05 and fold change ≥1.2-fold, with 199 upregulated and 248 downregulated proteins under the low-glucose condition (Fig. 3A).

To elucidate the functional roles of these proteins, we performed Kyoto Encyclopedia of Genes and Genomes (KEGG) pathway and WikiPathway enrichment analyses. Both analyses revealed that under a low-glucose condition, the pathways related to OXPHOS and the ETC were upregulated (Fig. 3B, C). Gene set enrichment analysis (GSEA) of KEGG and WikiPathways confirmed that proteins involved in mitochondrial OXPHOS and ETC pathways were significantly upregulated in PANC-1 cells under low-glucose conditions (Fig. 3D, E). According to criteria of *P*-value ≤ 0.01 and fold change ≥1.5, we identified seven mitochondrial-related proteins among the 45 proteins with upregulated expression in PANC-1 cells cultured in low-glucose conditions (Fig. 3F). These findings suggested that the PDAC cells reprogrammed energy metabolism toward mitochondrial OXPHOS and ETC under a condition of glycolytic suppression.

In general, acetyl-CoA enters the TCA cycle to generate NADH and FADH_2_, which are utilized by mitochondrial OXPHOS and ETC for ATP production [17]. The production of acetyl-CoA may be promoted in PDAC cells cultured under low-glucose conditions compared to cells cultured under normal glucose conditions. Additionally, we speculated that SP-2509 and OG-L002 decrease ATP levels by inhibiting the acetyl-CoA production in glycolysis-suppressed PDAC cells (Fig. 3G).

### SP-2509-decreased ATP production is not directly attributed to acetyl-CoA derived from the flux of pyruvate

**Fig. 4:**
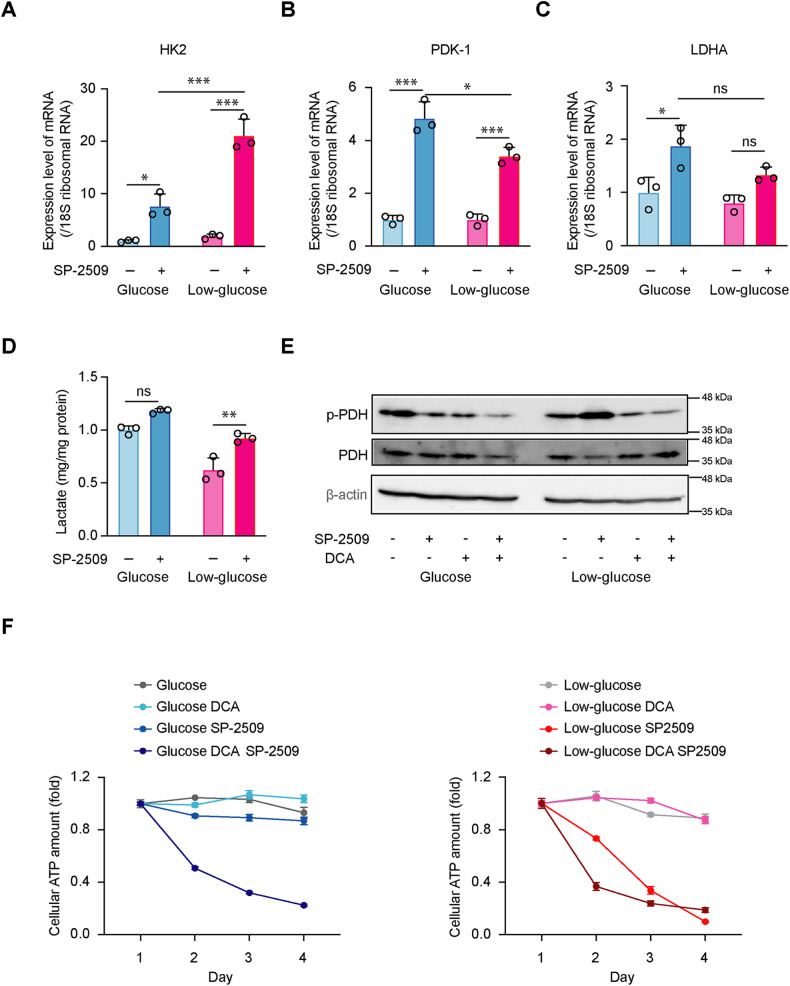
SP-2509-decreased ATP production is not directly attributed to acetyl-CoA derived from the flux of pyruvate. **A**–**C** PANC-1 cells were cultured in glucose or low-glucose medium with or without SP-2509 (10 µM) for 48 h. RT-qPCR analysis was performed to measure the mRNA levels of hexokinase II (HK2) (**A**), pyruvate dehydrogenase kinase 1 (PDK-1) (**B**), lactate dehydrogenase (LDHA) (**C**), and 18 S ribosomal RNA levels. Data are normalized against the levels of mRNA of each enzyme in cells cultured in glucose without SP-2509. Data are presented as mean ± SD for experiments performed in triplicate. Statistical analysis is based on two-way ANOVA followed by Tukey’s test for multiple comparisons. **D** PANC-1 cells were cultured in glucose or low-glucose medium with or without SP-2509 (10 µM) for 48 h. The amount of lactate released into the medium from the cells for the last 24 h and the protein in the cell lysates were measured. The ratio of total lactate amount/protein amount is used to indicate the level of lactate release. Data are normalized against the ratio of total lactate/total protein in cells cultured in glucose without SP-2509. Data are presented as mean ± SD for experiments performed in triplicate. Statistical analysis is based on two-way ANOVA followed by Tukey’s test for multiple comparisons. PANC-1 cells were cultured in glucose or low-glucose medium with SP-2509 (10 µM), dichloroacetate (DCA; 10 mM), or control conditions [dimethyl sulfoxide (DMSO) for SP-2509, MilliQ water for DCA] for 48 h (**E**) or for 1–4 d (**F**). Cell lysates were subjected to western blotting for phospho-PDH, PDH, and β-actin antibodies (**E**). The total intracellular ATP content was quantified each day. Data at each timepoint are normalized against the level at day 1. Data are presented as mean ± SD for experiments performed in triplicate (**F**). ***P < 0.001, **P < 0.01, *P < 0.05; ns not significant.

Acetyl-CoA can be produced from glucose, FAs, or acetate via various metabolic pathways [18]. Although SP-2509 is generally regarded as a suppressor of glycolysis via its effects on LSD1 [18, 19], we found that SP-2509 increased the mRNA levels of glycolytic enzymes (Fig. 4A–C) and promoted lactate production in PANC-1 cells (Fig. 4D), implying that SP-2509 enhances glycolysis in PDAC cells. PDH is a gatekeeper enzyme that connects glycolysis to the TCA cycle by converting pyruvate to acetyl-CoA [20]. PANC-1 cells cultured under low-glucose conditions showed enhanced PDH activity, compared to that of cells cultured under normal glucose conditions (Fig. 4E and Supplementary Fig. 3B). Notably, SP-2509 reversed the changes in PDH activity induced by glycolytic suppression (Fig. 4E and Supplementary Fig. 3B). Hence, we speculated that SP-2509 limits the flux of pyruvate. To investigate whether the limitation of the pyruvate flux is a major impact of the anticancer effect of SP-2509, we exposed PANC-1 cells treated with SP-2509 to dichloroacetate (DCA), an inhibitor of PDK, that negatively regulates PDH. As a result, exposure to SP-2509 alone resulted in a decrease in ATP levels following low-glucose conditions in PANC-1 cells, but co-exposure to SP-2509 and DCA failed to reverse the decrease (Fig. 4F). Therefore, acetyl-CoA levels derived from the flux of pyruvate do not appear to sufficiently explain how SP-2509 decreased ATP levels by PDAC cells under low-glucose conditions.

### SP-2509 and OG-L002 significantly increase intracellular lipid droplets (LD) accumulation independent of LSD1 function

**Fig. 5:**
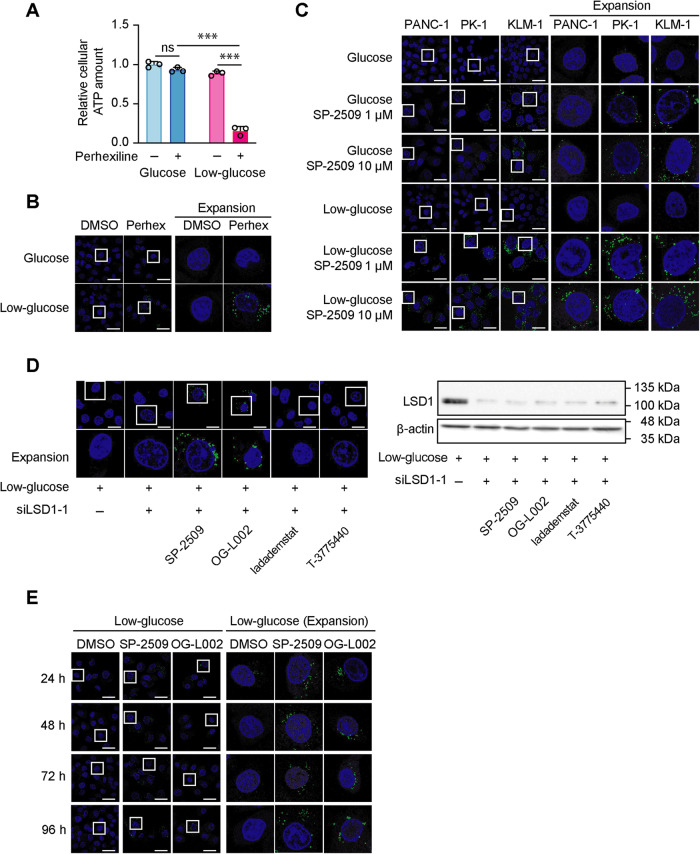
SP-2509 and OG-L002 significantly increase the accumulation of intracellular lipid droplets (LDs) independent of LSD1 function. **A** PANC-1 cells were cultured in glucose or low-glucose medium with or without perhexiline (10 µM) for 120 h prior to measuring the total intracellular ATP content. Data are normalized against the levels in PANC-1 cells cultured in glucose medium without perhexiline. Data are presented as mean ± SD for experiments performed in triplicate. Statistical analysis is based on two-way ANOVA followed by Tukey’s test for multiple comparisons. Staining of LDs (green); nuclei are shown in blue. Scale bar, 20 µm. Higher-magnification images are shown on the right side (**B**, **C**) or the downside (**D**). PANC-1 cells were cultured in glucose or low-glucose medium with or without perhexiline (10 µM) for 48 h (**B**), and PANC-1, PK-1, and KLM-1 cells were cultured in glucose or low-glucose medium with or without SP-2509 (1 and 10 µM) for 48 h (**C**). PANC-1 cells were transfected with si-control or si-LSD1-1 for 24 h and then the medium was replaced. Transfected cells were cultured in low-glucose medium with SP-2509 (10 µM), OG-L002 (50 µM), iadademstat (50 µM), T-3775440 (50 µM), or control agent for 48 h. The cell lysates were also subjected to western blotting for LSD1 and β-actin antibodies (**D**). **E** PANC-1 cells were cultured in low-glucose medium with or without SP-2509 (10 µM) or OG-L002 (50 µM) for 24 h, 48 h, 72 h and 96 h. LDs are shown in green and nuclei are shown in blue. Scale bar, 20 µm. Higher-magnification images are shown on the right side. ***P < 0.001; ns not significant.

Mitochondrial FA oxidation is another potentially significant source of acetyl-CoA in cells grown under glucose-deprived conditions [21]. To explore this potential mechanism of the observed anticancer effects, we treated the PDAC cells with perhexiline [22], which reduces the flux of FAs into the mitochondria by inhibiting CPT1 and CPT2. Perhexiline caused a marked decrease in intracellular ATP levels in PANC-1 cells grown under low-glucose conditions (Fig. 5A) and increased the accumulation of LDs (Fig. 5B). SP-2509 also induced the accumulation of LDs in all three PDAC cell lines, especially in cells cultured under conditions of glycolytic suppression (Fig. 5C and Supplementary Fig. 6). Depletion of LSD1 alone failed to induce the accumulation of LDs in PANC-1 cells cultured under low-glucose conditions, whereas LDs accumulation was observed in LSD1-depleted PANC-1 cells exposed to SP-2509 under low-glucose conditions (Fig. 5D and Supplementary Fig. 3C). We also exposed LSD1-depleted PANC-1 cells to the other LSD1 inhibitors, demonstrating that OG-L002, but not iadademstat or T-3775440, induced the accumulation of LDs under low-glucose conditions (Fig. 5D Supplementary Fig. 3C). Within only 24 h of exposure, SP-2509 induced the accumulation of LDs in PANC-1 cells cultured under low-glucose conditions (Fig. 5E). Taken together, these results suggested that SP-2509 and OG-L002 significantly increased the accumulation of intracellular LDs independent of LSD1 function. Moreover, there were no significant differences in the expression levels of the CPT1, CPT2, and acyl-CoA dehydrogenase genes between the dimethyl sulfoxide (DMSO; control)- and SP-2509-treated PANC-1 cells (Supplementary Fig. 7), implying that SP-2509 did not target mitochondrial FA uptake or oxidation enzymes.

### SP-2509 and OG-L002 specifically impair lipophagy in PDAC cells under glycolysis suppression

**Fig. 6:**
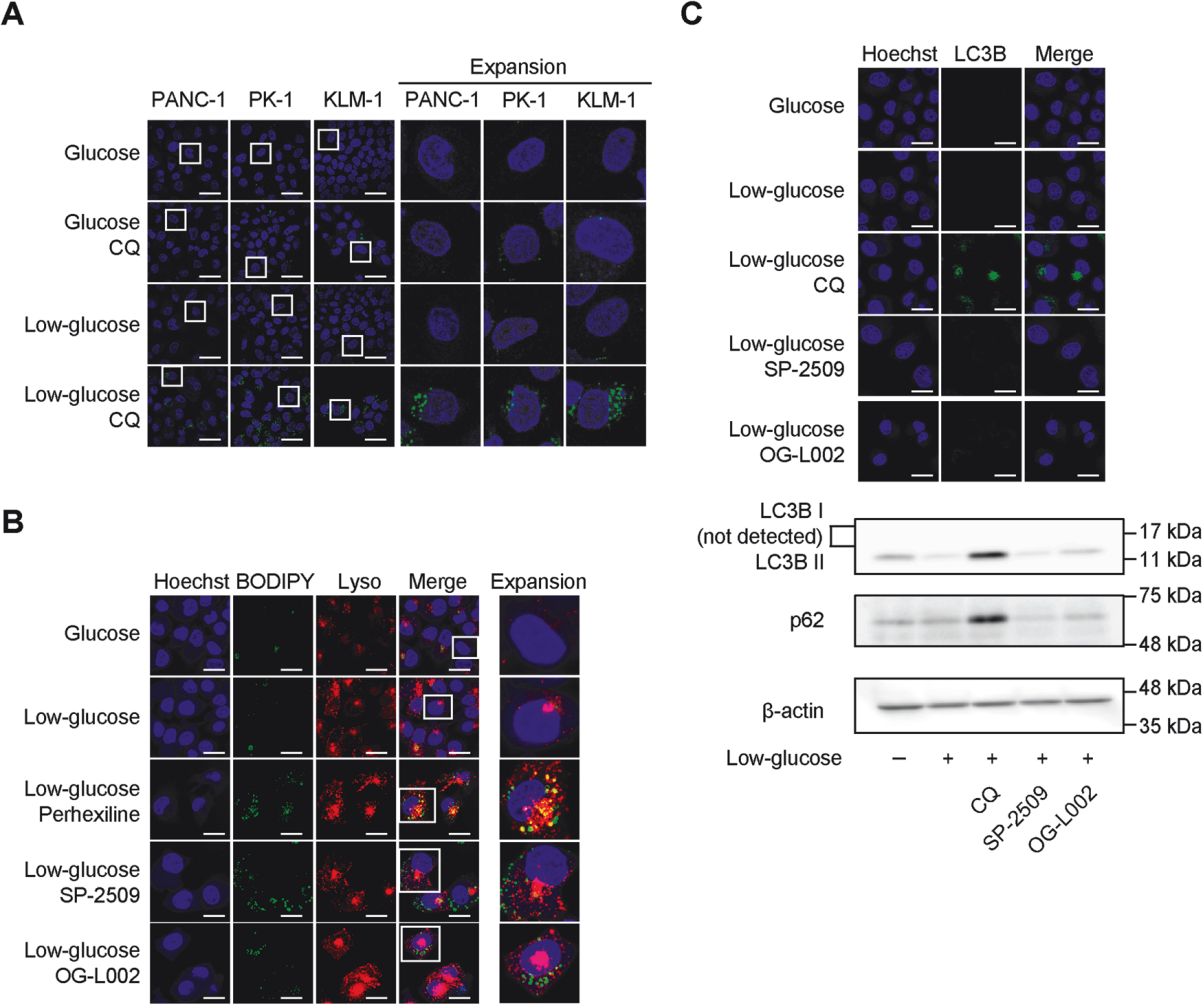
SP-2509 and OG-L002 specifically impair lipophagy in PDAC cells under glycolysis suppression. **A** PANC-1, PK-1, and KLM-1 cells were cultured in glucose or low-glucose medium with or without chloroquine (CQ; 10 µM) for 48 h. Lipid droplets (LDs) are shown in green and nuclei are shown in blue. Scale bar, 20 µm. Higher-magnification images are shown on the right side. **B**, **C** PANC-1 cells were cultured in low-glucose medium with or without perhexiline (10 µM), SP-2509 (10 µM) or OG-L002 (50 µM) for 48 h. LDs are shown in green, lysosomes (Lyso) are shown in red and nuclei are shown in blue. Higher-magnification images are shown on the right side. Scale bar, 20 µm. **B** The cell lysates were subjected to western blotting for LC3, p62, and β-actin antibodies. For immunofluorescence, LC3 is shown in green and nuclei are shown in blue. Scale bar, 20 µm (**C**).

Since our previous study indicated that suppression of glycolysis activated autophagy to maintain mitochondrial function and the survival of cancer cells [14], we next explored whether SP-2509 regulates LD accumulation through the autophagy pathway. Toward this end, we cultured glycolysis-suppressed PANC-1 cells with chloroquine (CQ), an autophagic flux inhibitor [23], which resulted in the significant accumulation of LDs (Fig. 6A). In general, FAs are stored in LDs upon reduction of nutrient levels in the tumor microenvironment, wherein they are hydrolyzed by autophagy in a process known as lipophagy [24]. To assess the impact of SP-2509 on the lipophagy process, we next performed dual staining of the lysosomes and LDs using Lysotracker (red) and BODIPY (green), respectively. Substantial co-localization of lysosomes and LDs was detected in cells cultured with perhexiline to inhibit mitochondrial FAs uptake (Fig. 6B), whereas minimal co-localized signals were detected in SP-2509- or OG-L002-cultured cells (Fig. 6B), suggesting that SP-2509 or OG-L002 inhibited the process before FAs uptake and LD fusion with lysosomes. Therefore, we found that neither SP-2509 nor OG-L002 interfered with autophagic flux and that bulk autophagy is not the mechanism contributing to the observed anticancer effects of these compounds under glycolysis suppression. (Fig. 6C and Supplementary Fig. 3D–E). Alternatively, these results suggest that SP-2509 and OG-L002 cause a disorder of LDs-related autophagosome fusion with lysosomes, an important step of lipophagy.

### The combination of 2DG and SP-2509 shows anti-tumoral effects

**Fig. 7:**
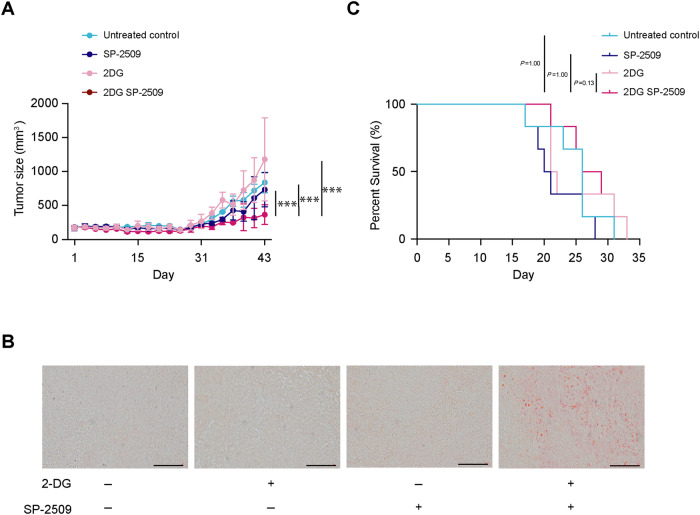
The combination of 2DG and SP-2509 shows anti-tumoral effects. **A** Tumor size of PANC-1 xenografts in CB.17SCID mice with the indicated treatments for 43 days. Data are presented as mean ± SD for experiments performed in triplicate. Statistical analysis is based on two-way ANOVA followed by Tukey’s test for multiple comparisons. **B** Tumors derived from KPC cells collected from C57BL/6 mice with indicated treatments for 7 days. Tumor sections were stained with Oil Red O to detect lipid droplets (LDs) in red; scale bar, 50 µm. **C** Kapan–Meier curve of C57BL/6 mice transplanted with KPC cells. Experiments were performed in sextuplicate. Statistical analysis is based on the log-rank (Mantel–Cox) test. ***P < 0.001.

Next, we investigated the in vivo antitumor effects of 2DG and SP-2509 in xenografts from CB.17SCID mice that were subcutaneously implanted with PANC-1 cells. Simultaneous administration of SP-2509 and 2DG significantly reduced the tumor size in mice compared to that of the other groups treated with each agent alone or the control (Fig. 7A), consistent with our in vitro results. Since CB.17SCID mice are severely immunodeficient and PANC-1 cells were subcutaneously implanted into the mice, we further employed another in vivo model of pancreatic cancer by implanting the KPC cell line into the pancreas of C57BL/6 mice. In vitro assessments confirmed that SP-2509 induced the accumulation of LDs and decreased the number of low glucose-cultured KPC cells (Supplementary Fig. 8). In mice, after one week of dual treatment with SP-2509 and 2DG, we observed a significant increase in the accumulation of intra-tumoral LDs (Fig. 7B) and the combination treatment showed a tendency of improved median survival outcomes compared to individual treatments, despite a lack of statistical significance (Fig. 7C). Taken together, these results indicated potential anti-tumor effects for the combination of 2DG and SP-2509 on PDAC.

## Discussion

Previous studies have implicated a role of LSD1 and LSD2 in tumor progression for various types of cancer, highlighting their potential as targets for drug discovery research [25–28]. SP-2509 has been suggested to slow down tumor growth by inhibiting LSD1 [19, 29], and its potential as a therapeutic agent has been explored in a phase I clinical trial including patients with Ewing sarcoma [30]. Here, we showed that SP-2509 has an anticancer effect on glycolysis-suppressed PDAC cells, whereas depletion of LSD1 and LSD2 did not influence PDAC cell growth, suggesting that SP-2509 targets other molecules in addition to its well-known effects on LSD1/LSD2. In support of this possibility, a previous study showed that SP-2509 decreased the number of acute myeloid leukemia cells with LSD1 knockout [31]. In addition to SP-2509, we also tested three other LSD1 inhibitors (OG-L002, iadademstat, and T-3775440), demonstrating that both SP-2509 and OG-L002 showed anticancer effects at concentrations far exceeding the IC50 for LSD1, whereas cell growth suppression was not observed with treatment of the possible maximum concentrations of iadademstat and T-3775440. Taken together, our data suggest the limited involvement of LSD1 as an anticancer target for PDAC and identify mechanisms underlying the effects of SP-2509 and OG-L002 may offer a new therapeutically important strategy for PDAC.

Abnormal glucose metabolism resulting from hyperglycemia or diabetes exacerbates pancreatic cancer [32]. Although antiglycolytic compounds have been explored as candidate therapies for pancreatic cancer cells, monotherapy with antiglycolytic compounds, such as 2DG, failed to show antitumor effects in mouse experiments and patients [33]. Our previous study showed that PANC-1 cells reprogrammed their metabolism to mitochondrial OXPHOS when cultured under conditions of glycolysis suppression [14]. Here, we found that inhibition of FA metabolism lowered ATP levels in PDAC cells cultured under glycolysis suppression, suggesting that FA metabolism plays an essential role in the survival of PDAC cells during glucose starvation. FA metabolism can sustain rapid cell proliferation and provides an essential energy source for cancer cells during metabolic stress [34], such as when there is reduced availability of serum-derived lipids in the tumor microenvironment [35]. Excessive FA uptake and oxidation are also associated with the development of liver cancer [36]. Upregulated FA synthesis, such as cholesterol synthesis, occurs in patients with glioblastoma and breast cancer [37]. Taken together, our results suggest that FA metabolism plays an essential role in helping PDAC cells survive under low-glucose conditions.

Released FAs are subsequently transported into the mitochondria where they undergo FA oxidation, producing ATP that supports tumor survival [38]. Here, we showed that SP-2509 did not induce a change in the expression of genes related to FA uptake and oxidation. Nevertheless, SP-2509 and OG-L002 significantly induced the accumulation of LDs in PDAC cells cultured under low-glucose conditions, implying that these compounds are more likely to target molecules related to FA storage. Lipid overload tends to provoke autophagy within cancer cells [37]. For example, cancer cells can employ LDs to modulate autophagy by providing lipid precursors for the formation of autophagic membranes [37]. In our previous study, we found that autophagy is activated in glycolysis-suppressed PANC-1 cells [14]. Here, we showed that both SP-2509 and OG-L002 did not affect bulk autophagy, while spatially segregated LDs from lysosomes. The reduced co-localization of LDs and lysosomes suggests the impairment of lipophagy [39]. The involvement of lipophagy in lipid turnover is crucial for both tumorigenesis and metastasis [40]. Poly-ubiquitination and LC3 may be important markers in the recognition of LDs for lipophagy [40]. However, the mechanism of lipophagy itself remains unclear [41]; therefore, it is currently not feasible to elucidate the in-depth mechanism by which SP-2509 and OG-L002 inhibit lipophagy in PDAC. Nevertheless, our results highlight the potential importance of targeting lipophagy in PDAC therapy and should motivate further research to investigate the underlying mechanism.

In conclusion, we propose a strategy to limit the proliferation of PDAC cells under glycolytic suppression. Specifically, FA metabolism is important for the survival of PDAC cells under glycolysis-suppressed conditions. FA metabolism plays an important role when intracellular energy metabolism is reprogrammed to OXPHOS under glycolysis suppression. Thus, perturbation of intracellular FAs metabolism causes the accumulation of LDs, resulting in anticancer effects in glycolysis-suppressed PDAC cells. Furthermore, we have indicated a potential link between LDs accumulation via lipophagy impairment and anticancer effects in glycolysis-suppressed PDAC cells. Determining the cancer-specific regulatory mechanism of lipophagy is therefore expected to expand the options of therapeutic strategies for PDAC.

## Materials and methods

### Reagents

2DG (FUJIFILM WAKO, Tokyo, Japan), CQ (Sigma-Aldrich, St. Louis, MO, USA), and iadademstat (ORY-1001) 2HCl (Selleckchem, Houston, TX, USA) were dissolved in MilliQ water. SP-2509 (Merck, Darmstadt, Germany), OG-L002 (Selleckchem), T-3775440 HCl (Selleckchem), DCA (Tokyo Kasei Corp., Tokyo, Japan), perhexiline maleate salt (Cayman Chemical Company, Ann Arbor, MI, USA), and oligomycin (Sigma-Aldrich) were dissolved in DMSO (Nacalai Tesque Inc., Kyoto, Japan).

### Cell culture

PANC-1, PK-1, and KLM-1 cells were purchased from the RIKEN Cell Engineering Division (RIKEN BioResource Research Center, Tsukuba, Japan). The cells were maintained in RPMI-1640 medium (Nacalai Tesque) supplemented with 10% fetal bovine serum (Life Technologies, Grand Island, NY, USA) and antibiotics (Nacalai Tesque). The KPC cell line (established from mice with a C57BL/6 background) was purchased from CancerTools (London, UK) and maintained in Dulbecco’s modified Eagle medium (Nacalai Tesque) supplemented with 10% fetal bovine serum and antibiotics. All cells were cultured at 37 °C in a humidified chamber with 5% CO_2_. PANC-1 cells were cultured in medium supplemented with normal glucose (2 g/L), low glucose (0.2 g/L), or galactose (2 g/L), or low levels of glucose (0.2 g/L). PK-1 and KLM-1 cells were cultured in medium supplemented with glucose at 2 g/L or 0.2 g/L. KPC cells were cultured with 4.5 g/L or 0.45 g/L glucose.

### Metabolic compound screening

An Anti-Cancer Metabolism Compound library (96-well, catalog number L2130) was purchased from TargetMol (Wellesley Hills, MA, USA). PANC-1 cells were cultured in glucose (2 g/L), galactose (2 g/L), or low-glucose (0.2 g/L) medium with 130 different types of metabolic compounds for 48 h. To assess the cell number, the cells were incubated with 0.5 mg/mL 3-(4,5-dimethylthiazol-2-yl)-2,5-diphenyltetrazolium bromide (MTT; Dojindo Laboratory, Kumamoto, Japan) for 2 h. After incubation, the formazan crystals produced were dissolved in 200 μL DMSO. The absorbance of the resulting solution was measured at 540 nm to determine the amount of MTT-formazan formed.

### Measurement of intracellular ATP concentration

The intracellular concentration of ATP was assessed using the CellTiter-Glo Luminescent Cell Viability Assay (Promega, Madison, WI, USA) according to the manufacturer’s protocol.

### Western blotting

Cells were lysed on ice in lysis buffer [phosphate-buffered saline (PBS), (pH 7.4), containing 1% Triton X-100] (Nacalai Tesque) and a protease inhibitor cocktail (Roche, Mannheim, Germany). Equal amounts of protein from each sample were heated to 95 °C for 5 min, separated by sodium dodecyl sulfate-polyacrylamide gel electrophoresis, and then transferred to a polyvinylidene difluoride membrane (Millipore, Berlin, Germany). The membranes were blocked with 5% bovine albumin (Nacalai Tesque) and 1% or 5% non-fat dry milk (Cell Signaling Technology, Beverly, MA, USA) at 25 °C for 1 h. The membranes were then probed overnight with primary antibodies specific for H3K4me3 (Cell Signaling Technology), LC3B (D11) (Cell Signaling Technology), LSD1 (C69G12) (Cell Signaling Technology), phospho-PDH α1 (Ser293) (Cell Signaling Technology), PDH (Cell Signaling Technology), p62 (MBL Life Science, Nagoya, Japan), or β-actin (Sigma-Aldrich). Immunolabeled proteins were detected using horseradish peroxidase-conjugated secondary antibodies (GE Healthcare, Buckinghamshire, UK) and ECL Prime detection reagents (GE Healthcare). The signals were visualized using an ImageQuant LAS 4000 (version 1.3) system (GE Healthcare).

### Transfection of small interfering RNA (siRNA)

The cells were transfected with siRNA oligonucleotides (Japan Bio Services Co. Ltd., Saitama, Japan) at final concentrations of 100 nM using Lipofectamine 2000 (Invitrogen, Waltham, MA, USA). Details of oligonucleotide sequences are presented in Supplementary Table 2.

### RNA isolation and quantitative real-time PCR

Total RNA was isolated from PANC-1 cells using Sepasol-RNA I reagent (Nacalai Tesque) and reverse-transcribed using ReverTra Ace qPCR RT Master Mix (TOYOBO, Osaka, Japan). The resulting cDNA was mixed with THUNDERBIRD quantitative real-time PCR mix (TOYOBO). The mixture was then subjected to quantitative real-time PCR. Primers used in this study are listed in Supplementary Table 3. The cycling conditions were as follows: 95 °C for 60 s, followed by 40 cycles (50 cycles for long-chain acyl-CoA dehydrogenase) of 95 °C for 10 s and 60 °C for 60 s. Relative mRNA expression levels were calculated after normalization against the levels of 18 S ribosomal RNA or β-actin.

### Measurement of cellular lactate

Cellular lactate levels were measured using a Lactate Assay Kit-WST (Dojindo Laboratory).

### Proteomic analyses based on nano liquid chromatography-tandem mass spectrometry (NanoLC-MS/MS) and bioinformatics analysis

PANC-1 cells were cultured in glucose or low-glucose medium for 6 days. The cells were then lysed with ice-cold lysis buffer [PBS (pH 7.4) containing 1% Triton X-100, protease inhibitor, and phosphatase inhibitor cocktail (Roche)]. Proteins were precipitated from cell lysates using the ProteoExtract Protein Precipitation Kit (Millipore), according to the manufacturer’s guidelines. Precipitated proteins were dissolved in approximately 10 µL of 50 mM ammonium bicarbonate buffer (pH 8.1) containing 0.1% RapiGest SF (Waters Corporation, Milford, MA, USA). Total protein concentrations were determined using Pierce™ 660 nm protein assay reagent (Thermo Fisher Scientific, Waltham, MA, USA), including Ionic Detergent Compatibility Reagent (Thermo Fisher Scientific). To each 0.5 mL tube, 10 µg of protein was aliquoted and diluted with 25.5 µL of 50 mM ammonium bicarbonate buffer (pH 8.1) containing 0.1% RapiGest SF and 1.5 µL of dithiothreitol (100 mM in distilled water; Nacalai Tesque). The solution was heated at 60 °C for 30 min. After cooling to 25 °C, 3 µL of iodoacetamide (100 mM in distilled water; FUJIFILM WAKO) was added to the solution (final concentration of 10 mM), and the tubes were incubated at room temperature for 30 min in the dark. The samples were then digested with trypsin (mass spectrometry-grade, Promega; protein/enzyme = 20/1, w/w) at 37 °C overnight. The solution was quenched with 10% trifluoroacetic acid (pH < 3) and incubated at 37 °C for 30 min. After centrifugation (13,300 × *g*, 10 min, 25 °C), the solution was desalted using GL-Tip SDB (GL Science, Tokyo, Japan) according to the manufacturer’s instructions. The eluate was dried *in vacuo* and dissolved in distilled water containing 2% MeCN and 0.1% formic acid.

For proteomic analysis, nanoLC-MS/MS analyses was performed on an Ultimate 3000 RSLCnano system (Thermo Fisher Scientific) coupled to a Q Exactive hybrid quadrupole-Orbitrap mass spectrometer (Thermo Fisher Scientific) equipped with a nano-electrospray ionization source. The nanoLC system was equipped with a trap column (Thermo Fisher Scientific) and analytical column (Nikkyo Technos, Tokyo, Japan). Peptide separation was performed using a 90 min gradient between water containing 0.1% formic acid (mobile phase A) and acetonitrile containing 0.1% formic acid (mobile phase B) at a flow rate of 300 nL/min. The elution was set as follows: 0–3 min, 2% B; 3–63 min, 2–40% B; 93–95 min, 40–95% B; 95–105 min, 95% B; 105–107 min, 95–2% B; 107–120 min, 2% B. The mass spectrometer was operated in data-dependent acquisition mode. MS parameters were set as follows: spray voltage, 2.0 kV; capillary temperature, 275 °C; S-lens RF level, 50; scan type, full MS; scan range, *m/z* 350–1500; resolution, 70 000; polarity, positive; automatic gain control target, 3 × 10^6^; and maximum injection time, 100 ms. MS/MS parameters were set as follows: resolution, 17 500; automatic gain control target, 1 × 10^5^; maximum injection time, 60 ms; normalized collision energy, 27; dynamic exclusion, 15 s; loop count, 10; isolation window, 1.6 *m*/*z*; and charge exclusion, unassigned, 1, 8, >8. Measurements were performed in duplicate for each sample and all replicated data were merged and used for quantitative analysis.

Protein identification and relative quantitation were performed using the Proteome Discoverer 2.4 SP1 system (Thermo Fisher Scientific). Search parameters were as follows: search engine, Sequest HT; protein database, SwissProt (*Homo sapiens*); enzyme name, trypsin (full); dynamic modification, oxidation (methionine, +15.99 Da); static modification, carbamidomethyl (cysteine, +57.02 Da); precursor mass tolerance of 10 ppm; and fragment mass tolerance of 0.02 Da. The label-free quantification parameters for the detected peptides were set as follows: precursor quantification, precursor abundance based on area, normalization mode, and total peptide amount.

Gene symbols of 199 proteins upregulated under low-glucose conditions were used for pathway enrichment analysis in g:Profiler (https://biit.cs.ut.ee/gprofiler/gost). The raw data are presented in Supplementary Table 4. The abundance of proteins under glucose or low-glucose conditions was used for analysis with GSEA 4.3.2 with the following settings: number of permutations = 1000, permutation type = gene set, enrichment statistics = weighted, metric for ranking genes = ratios of classes.

### Immunofluorescence

The cells were seeded in 35-mm glass-bottom dishes (MATSUNAMI, Japan) and left overnight to attach. After CQ or SP-2509 treatment, cells were incubated with LysoTracker (1 μM) (Thermo Fisher Scientific, Hanover Park, IL, USA) for 1 h. Subsequently, the cells were fixed in 4% paraformaldehyde for 10 min. Fixed cells were stained with 1 μg/mL BODIPY-493-/503 (Thermo Fisher Scientific) and 5 μg/mL Hoechst 33342 for 30 min at 37 °C. Immunofluorescence staining of LC3 was performed as previously described [14]. Nuclei were stained with 5 μg/mL Hoechst 33342. All samples were imaged using a Carl Zeiss LSM780 laser-scanning confocal microscope (Prenzlauer, Berlin, Germany). Images of five random fields of vision from each group were captured and analyzed using Image J.

### Animals and treatment protocols

Female CB.17SCID mice (12 pups) and C57BL/6 mice (36 pups) aged 6 weeks were purchased from The Jackson Laboratory (Kanagawa, Japan) and maintained in an experimental animal facility at Chiba University (Chiba, Japan). For CB.17SCID mice, each mouse was subcutaneously administered 1.5 × 10^6^ PANC-1 cells suspended in 50 µL serum-free RPMI-1640 and 50 µL Cultrex^TM^ Basement Membrane Extract, Type3, PathClear^TM^ (bio-techne, Minneapolis, MN, USA). After 7 days, the tumor volume was over 100 mm^3^ and the mice were randomly divided into the following four treatment groups: untreated control, 2DG alone, SP-2509 alone, and a combination of 2DG and SP-2509. SP-2509 (0.5 mg/mouse) was administered intraperitoneally to the mice twice per week and 2DG (10 mg/mouse) was administered intraperitoneally three times per week. Tumor volumes were calculated using the following standard formula: width^2^ × length × 0.52 [42], which were measured every 2-3 days using Mitutoyo digital calipers (Mitutoyo, Tokyo, Japan). The tumor sizes were controlled so that their volumes did not exceed 2000 mm^3^. For C57BL/6 mice, the pancreas was orthotopically injected with 5 × 10^5^ KPC cells suspended in 50 µL Hanks’ Buffered Salt Solution buffer. After 7 days, the mice were randomly divided into the following four treatment groups: untreated control, 2DG alone, SP-2509 alone, and a combination of 2DG and SP-2509. SP-2509 (0.5 mg/mouse) was administered intraperitoneally to the mice twice per week, and 2DG (10 mg/mouse) was administered intraperitoneally three times per week.

### Oil Red O staining

Oil Red O staining was conducted as previously described [43]. Samples were imaged using ECLIPSE Ci-L plus Upright Microscope (NIKON, Tokyo, Japan).

### Statistical analysis

All data are presented as mean ± SD of at least three independent experiments, unless indicated otherwise. Statistical analysis was performed using an unpaired Student’s *t* test, log-rank (Mantel–Cox) test, one-way or two-way ANOVA followed by Tukey’s test. A *P* value < 0.05 was considered statistically significant. All analyses were conducted using GraphPad Prism version 9 (Dotmatics, Boston, MA, USA).

### Supplementary information

Supplementary Figures

Supplementary Figure 3

Supplementary Table 1

Supplementary Table 2

Supplementary Table 3

Supplementary Table 4

## Data Availability

Additional data and materials supporting the findings of this study are available upon request from the corresponding authors. Supplementary information is available at https://www.nature.com/cddis/.
