# Supplementary material for: Potential role of lipophagy impairment for anticancer effects of glycolysis-suppressed pancreatic ductal adenocarcinoma cells
Source: Cell Death Discov. 2024 Apr 5;10:166. doi: 10.1038/s41420-024-01933-4 (PMC10997792; doi:10.1038/s41420-024-01933-4)
Supplement: Supplementary file 1 — Supplementary Figures [file 41420_2024_1933_MOESM1_ESM.pdf]

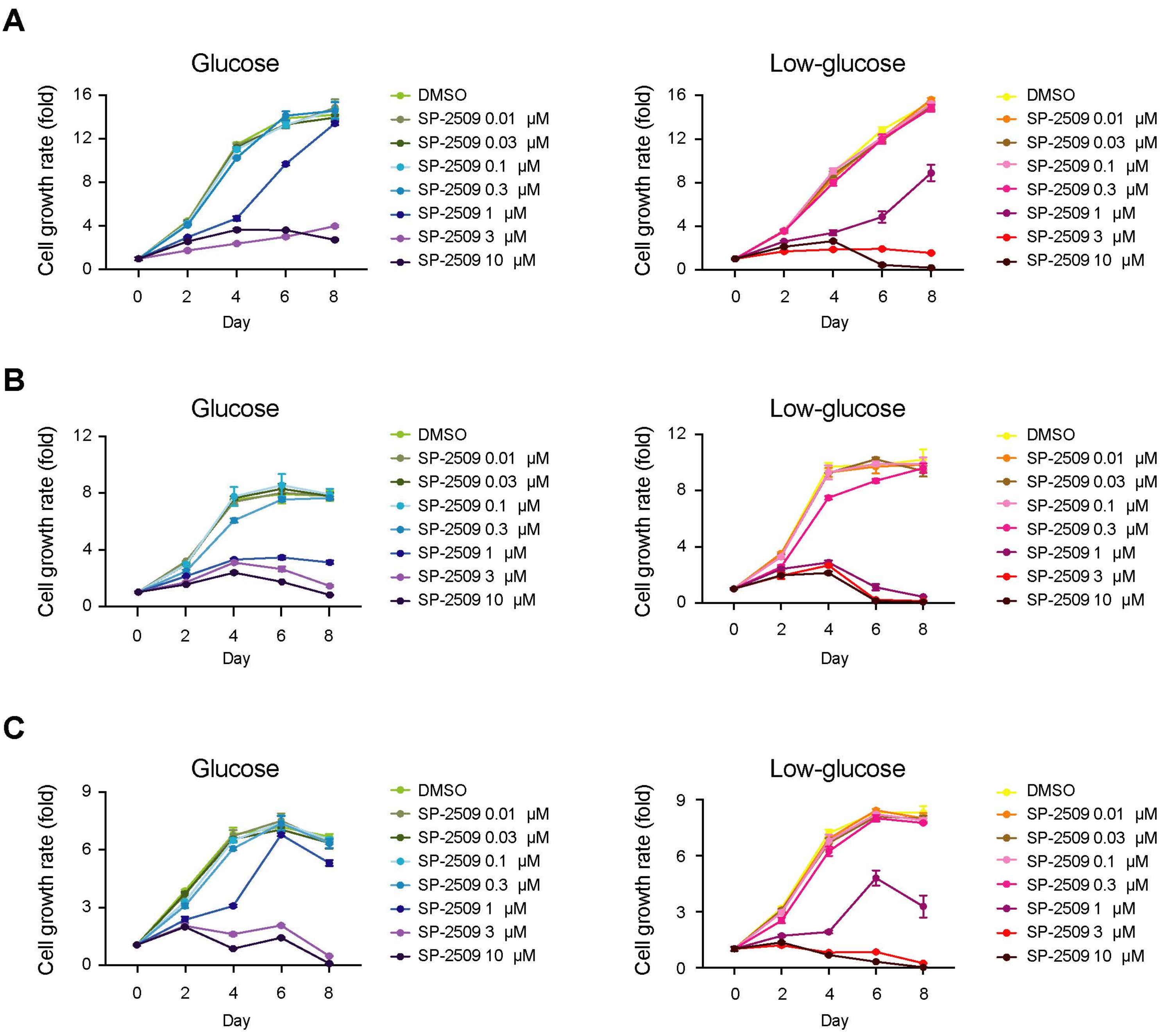

Supplementary Fig. 1. Effects of different concentrations of SP-2509 on the growth of PDAC cells. A–C, PANC-1 (**A**), PK-1 (**B**), and KLM-1 (**C**) cells were cultured in glucose or low-glucose medium with or without SP-2509 (0.01–10 μM) for eight days. The cell number was determined via an MTT assay. The level of produced formazan crystals was quantified each day. Data at each timepoint are normalized against day 0. Data are presented as mean ± SD for experiments performed in triplicate.

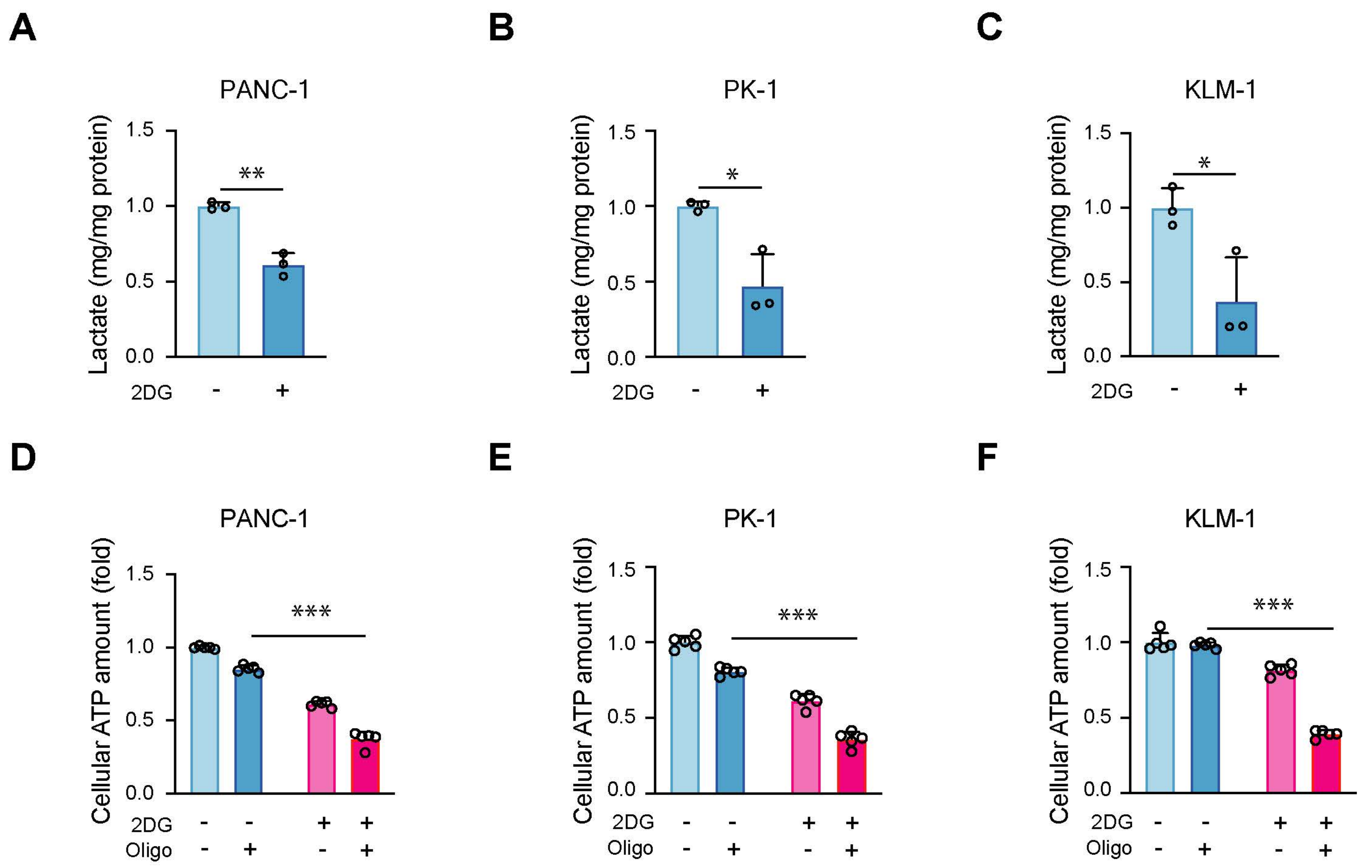

Supplementary Fig. 2. PDAC cells reprogrammed to mitochondrial OXPHOS when cultured with 2-deoxyglucose (2DG). A–C, PANC-1 (**A**), PK-1 (**B**), and KLM-1 (**C**) cells were cultured with or without 2DG (10 mM for PANC cells, 1 mM for PK-1 and KLM-1 cells) for 48 h. The amount of lactate released into the medium from the cells during the last 24 h and the amount of protein in the cell lysates were measured. The ratio of total lactate amount/protein amount is used to indicate the level of lactate release. Data are normalized against the levels in cells cultured without 2DG. Data are presented as mean ± SD for experiments performed in triplicate. Statistical analysis is based on unpaired Student' s test; ** $P < 0.01$, * $P < 0.05$. **D–F**, PANC-1 (**D**), PK-1 (**E**), and KLM-1 (**F**) cells treated with or without 2DG (10 mM for PANC-1 cells, 1 mM for PK-1 and KLM-1 cells) were cultured with or without oligomycin (Oligo; 20 ng/mL) for 48 h and then the intracellular ATP content was quantified. Data are normalized against the levels in cells cultured without 2DG or Oligo. Data are presented as mean ± SD for experiments performed in quintuple. Statistical analysis is based on two-way ANOVA followed by Tukey' s test for multiple comparisons. *** $P < 0.001$.

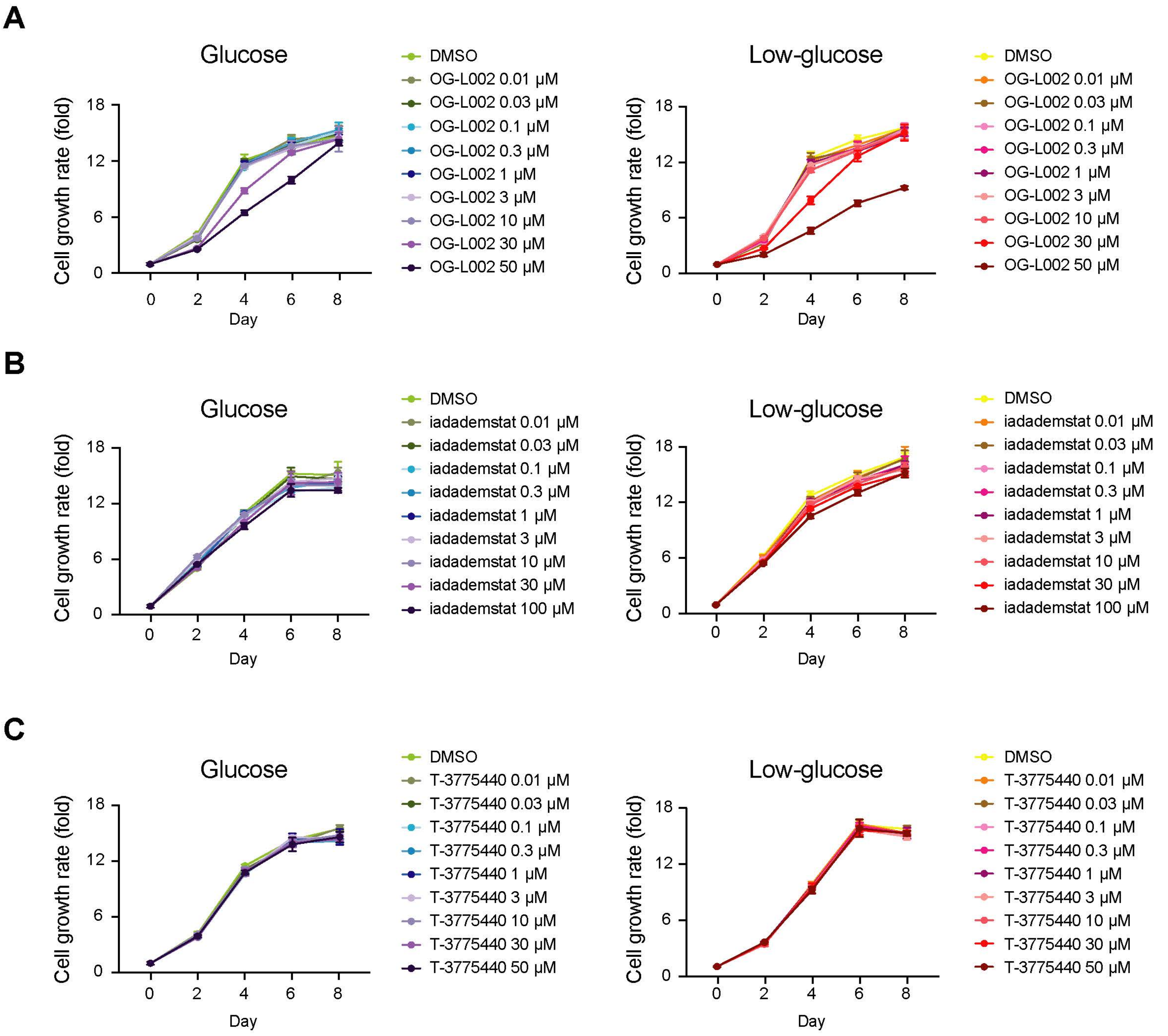

Supplementary Fig. 4. Effects of LSD1 inhibitors at different concentrations on the growth of PANC-1 cells. A–C, PANC-1 cells were cultured in glucose or low-glucose medium with or without OG-L002 (0.01–50 μM) (**A**), iadademstat (0.01–100 μM) (**B**), or T-3775440 (0.01–50 μM) (**C**) for eight days. The cell number was determined via an MTT assay. The level of produced formazan crystals was quantified each day. Data at each timepoint are normalized against the day 0 level. Data are presented as mean ± SD for experiments performed in triplicate.

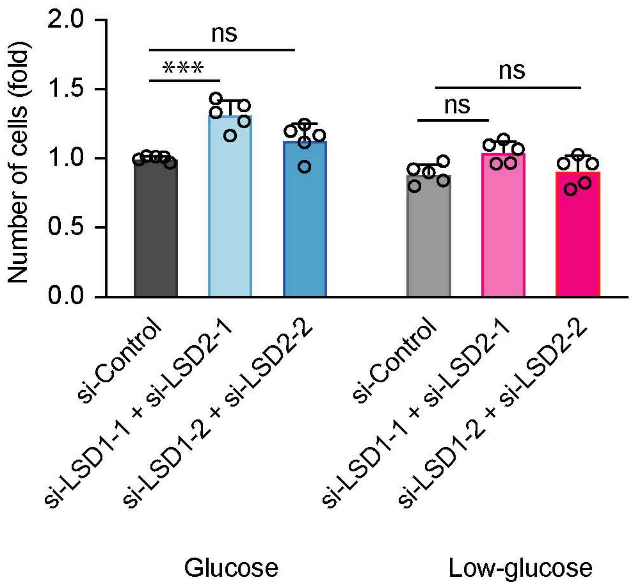

Supplementary Fig. 5. LSD1 and LSD2 dual depletion did not reduce the number of PDAC cells in a glucose or low-glucose condition. PANC-1 cells were transfected with si-Control, si-LSD1, or si-LSD2 for 24 h and then the medium was replaced. Transfected cells were cultured with glucose or low-glucose medium for another 48 h. The number of cells was determined by a 3-(4,5-dimethylthiazol-2-yl)-2,5-diphenyltetrazolium bromide (MTT) assay. Data are normalized against the level in si-Control-transfected cells cultured in glucose medium. Data are presented as mean ± SD for experiments performed in quintuple. Statistical analysis is based on two-way ANOVA followed by Tukey's test for multiple comparisons. *** $P < 0.001$; ns, not significant.

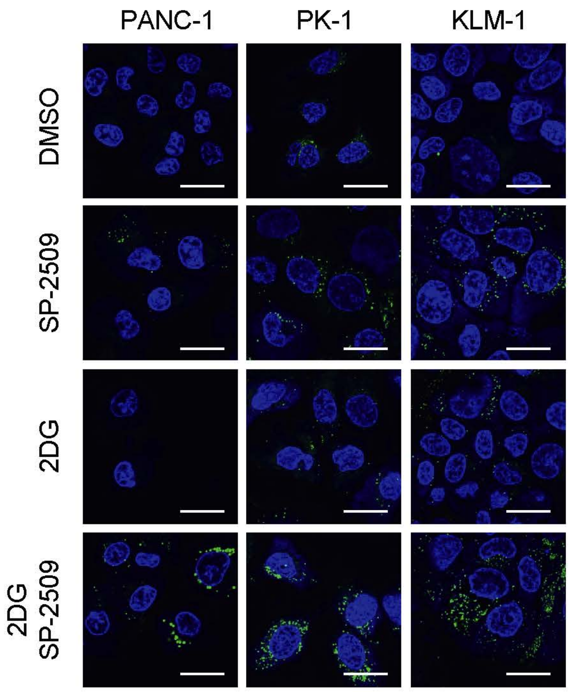

Supplementary Fig. 6. SP-2509 significantly increased intracellular lipid-droplet (LD) accumulation, especially in glycolysis-suppressed PDAC cells. PANC-1, PK-1, and KLM-1 cells treated with or without 2-deoxyglucose (2DG; 10 mM for PANC-1 cells, 1 mM for PK-1 and KLM-1 cells) were cultured with or without SP-2509 (10 μM) for 48 h. LDs are shown in green and nuclei are shown in blue. Scale bar, 20 μm.

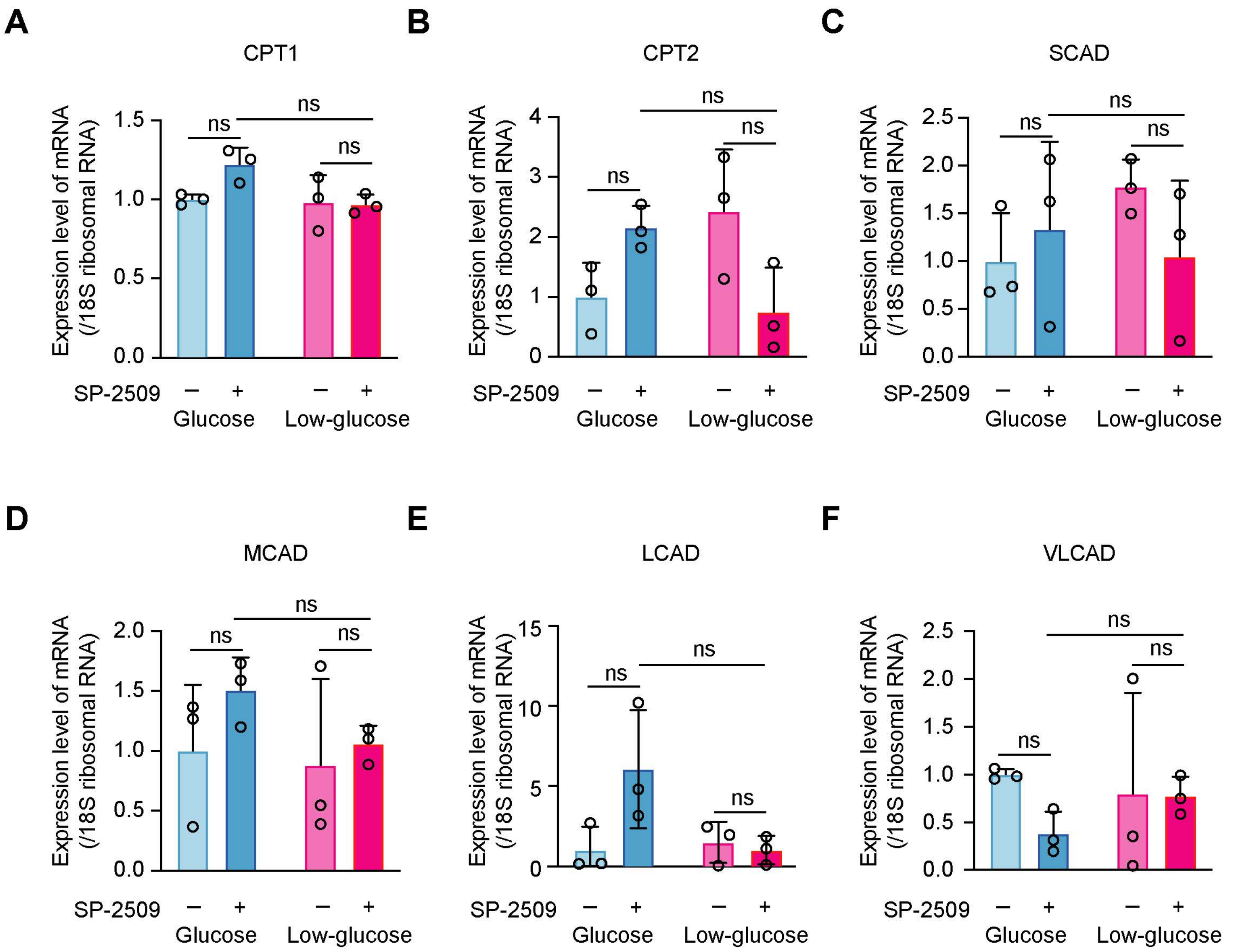

Supplementary Fig. 7. SP-2509 did not alter the mRNA levels of fatty acid beta-oxidation factors in mitochondria. PANC-1 cells were cultured in glucose or low-glucose medium with or without SP2509 (10 μM) for 48 h. RT-qPCR analysis was performed to measure the mRNA levels of the CPT1 (**A**), CPT2 (**B**), short chain acyl-CoA dehydrogenase (SCAD) (**C**), medium chain acyl-CoA dehydrogenase (MCAD) (**D**), long chain acyl-CoA dehydrogenase (LCAD) (**E**), and very long chain acyl-CoA dehydrogenase (VLCAD) (**F**) genes; 18S ribosomal RNA levels served as the reference. Data are normalized against the levels of the corresponding molecules in cells cultured in glucose medium without SP2509. Data are presented as mean ± SD for experiments performed in triplicate. Statistical analysis is based on two-way ANOVA followed by Tukey' s test for multiple comparisons. ns, not significant.

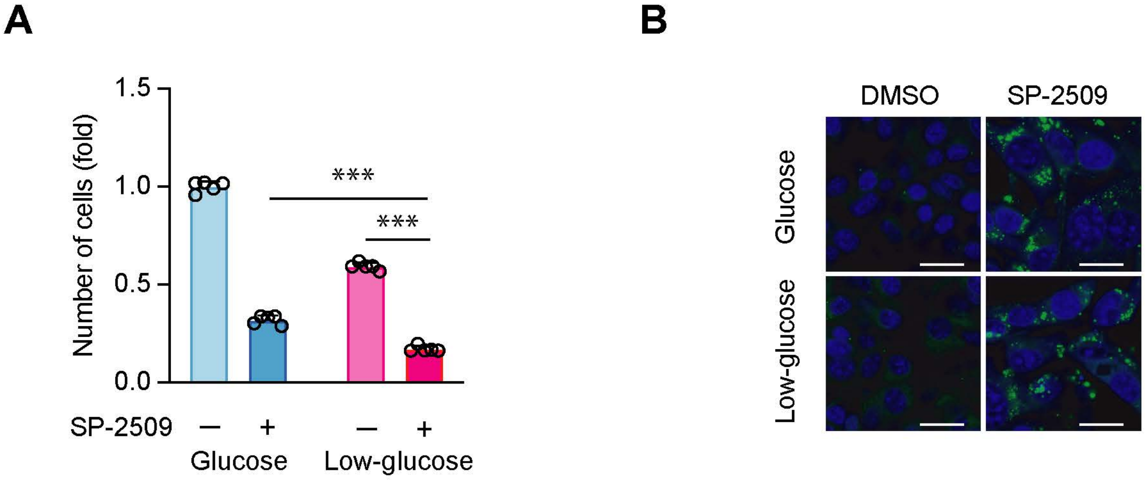

Supplementary Fig. 8. SP-2509 reduced the accumulation of lipid droplets (LDs) in glycolysis-suppressed KPC cells. **A** and **B**, KPC cells were cultured in glucose or low-glucose medium with or without SP-2509 (10 μM) for 48 h. The cell number was determined via an MTT assay. Data are normalized against the levels in KPC cells cultured in glucose medium without SP-2509. Data are presented as mean ± SD for experiments performed in quintuple (**A**). LDs are shown in green and nuclei are shown in blue. Scale bar, 20 μm. (**B**). *** $P < 0.001$.
