# Supplementary material for: Potential role of lipophagy impairment for anticancer effects of glycolysis-suppressed pancreatic ductal adenocarcinoma cells
Source: Cell Death Discov. 2024 Apr 5;10:166. doi: 10.1038/s41420-024-01933-4 (PMC10997792; doi:10.1038/s41420-024-01933-4)
Supplement: Supplementary file 2 — Supplementary Figure 3 [file 41420_2024_1933_MOESM2_ESM.pptx]

## Slide 1
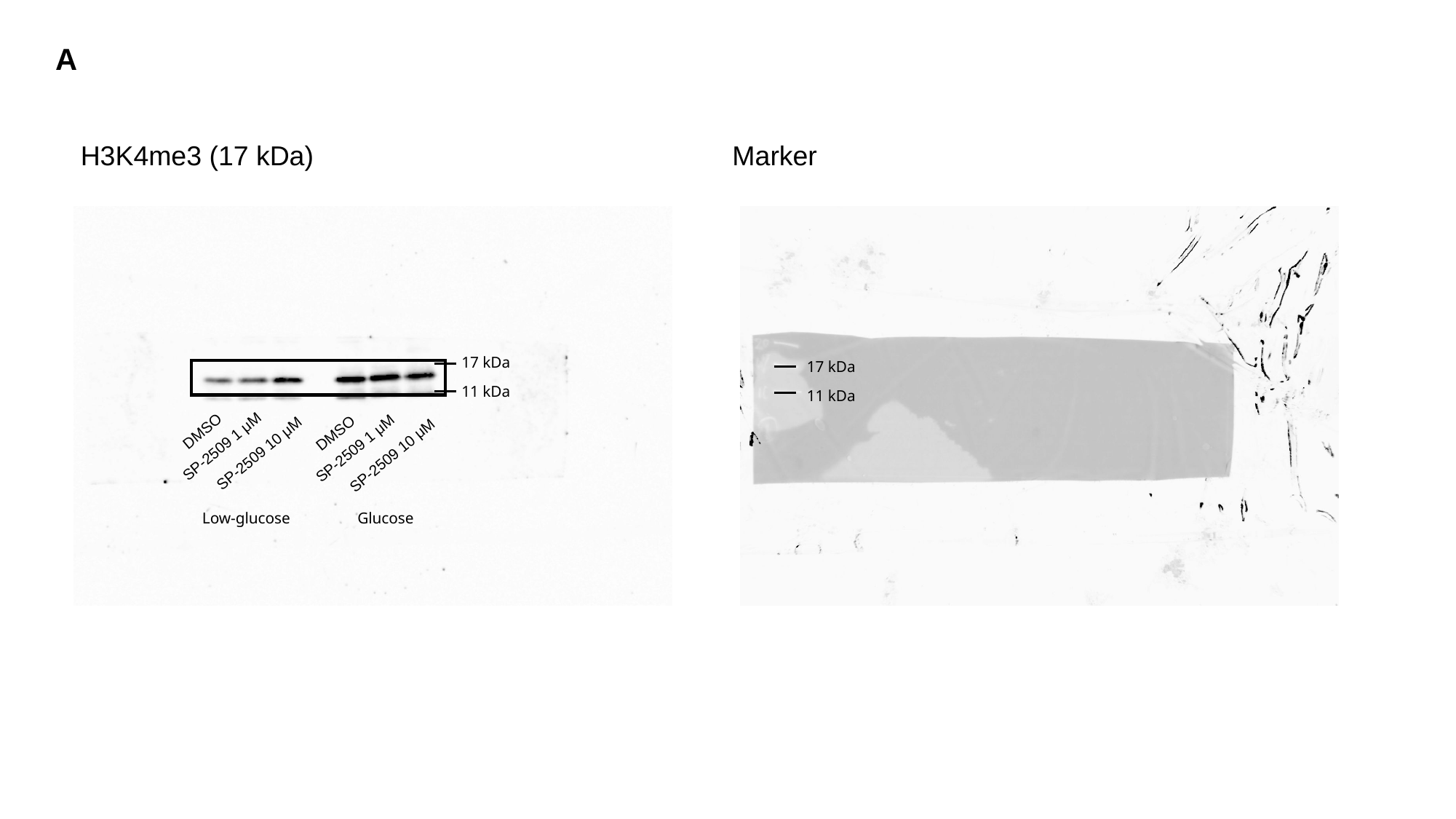

A
H3K4me3 (17 kDa)
Marker
17 kDa
17 kDa
11 kDa
11 kDa
DMSO
DMSO
SP-2509 1 μM
SP-2509 1 μM
SP-2509 10 μM
SP-2509 10 μM
Low-glucose
Glucose

## Slide 2
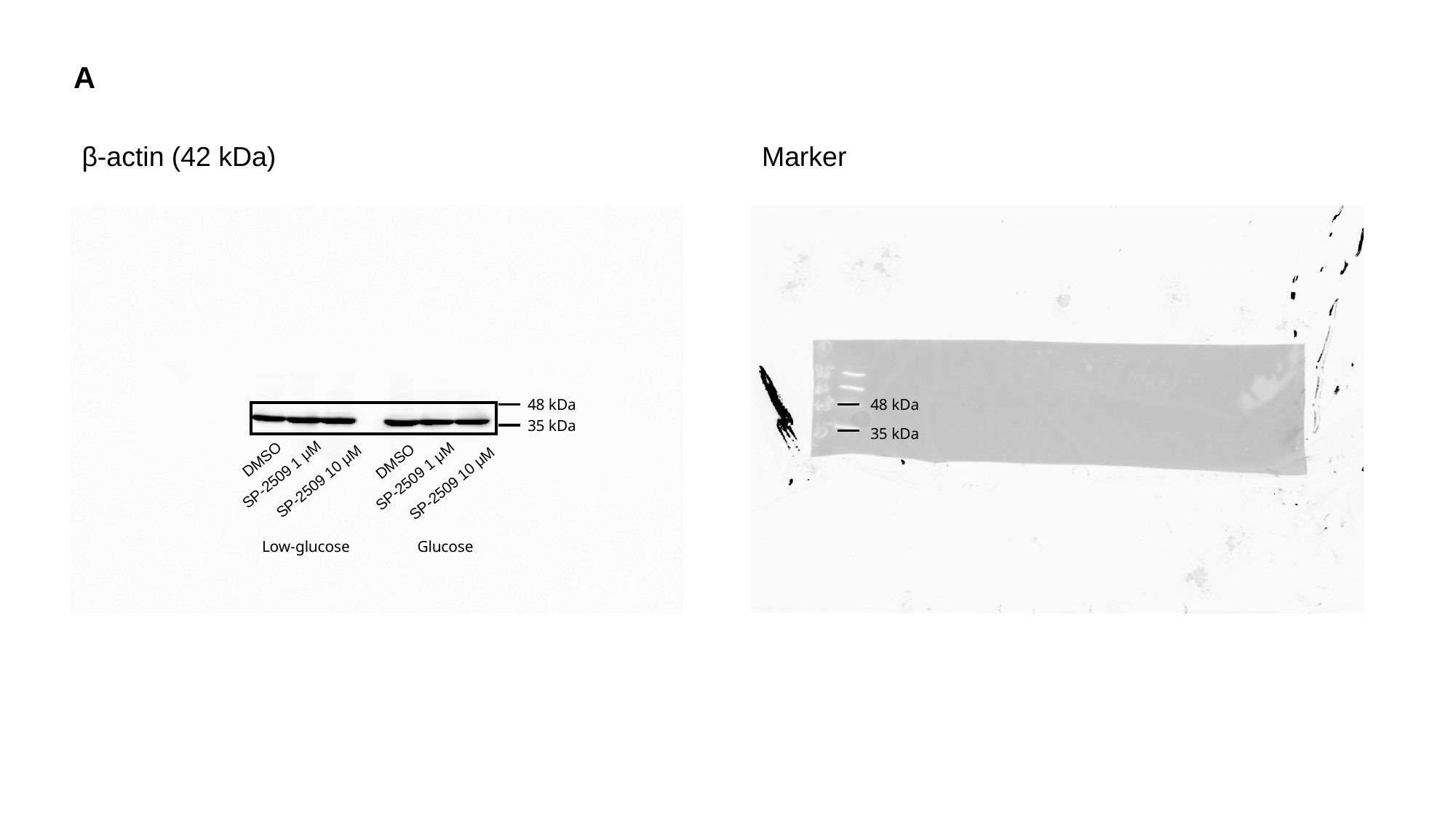

A
β-actin (42 kDa)
Marker
48 kDa
48 kDa
35 kDa
35 kDa
DMSO
DMSO
SP-2509 1 μM
SP-2509 1 μM
SP-2509 10 μM
SP-2509 10 μM
Low-glucose
Glucose

## Slide 3
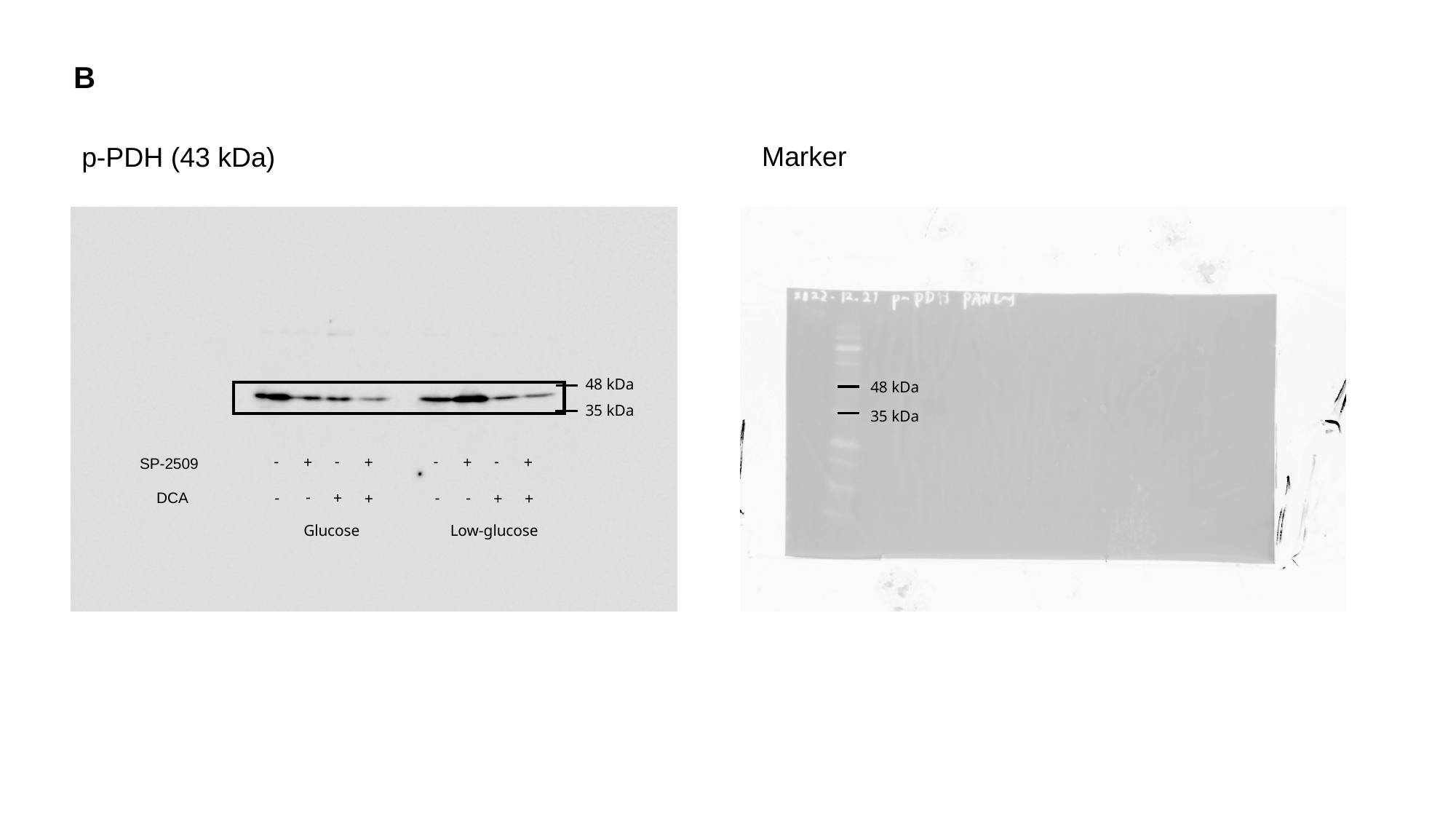

B
Marker
p-PDH (43 kDa)
48 kDa
48 kDa
35 kDa
35 kDa
-
-
-
-
+
+
+
+
SP-2509
-
-
DCA
-
+
-
+
+
+
Low-glucose
Glucose

## Slide 4
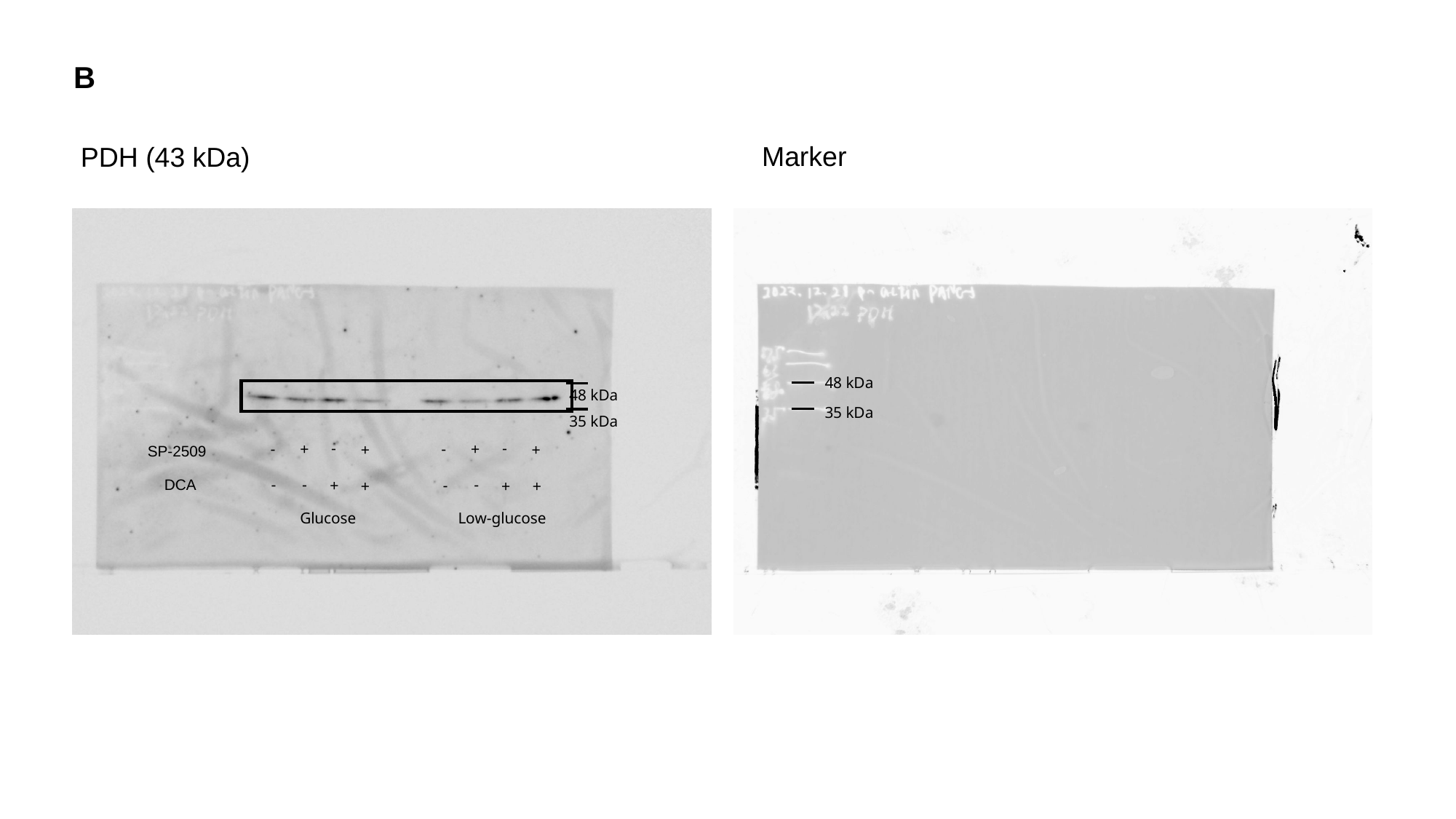

B
Marker
PDH (43 kDa)
48 kDa
48 kDa
35 kDa
35 kDa
-
-
-
-
+
+
+
+
SP-2509
-
-
DCA
-
+
-
+
+
+
Low-glucose
Glucose

## Slide 5
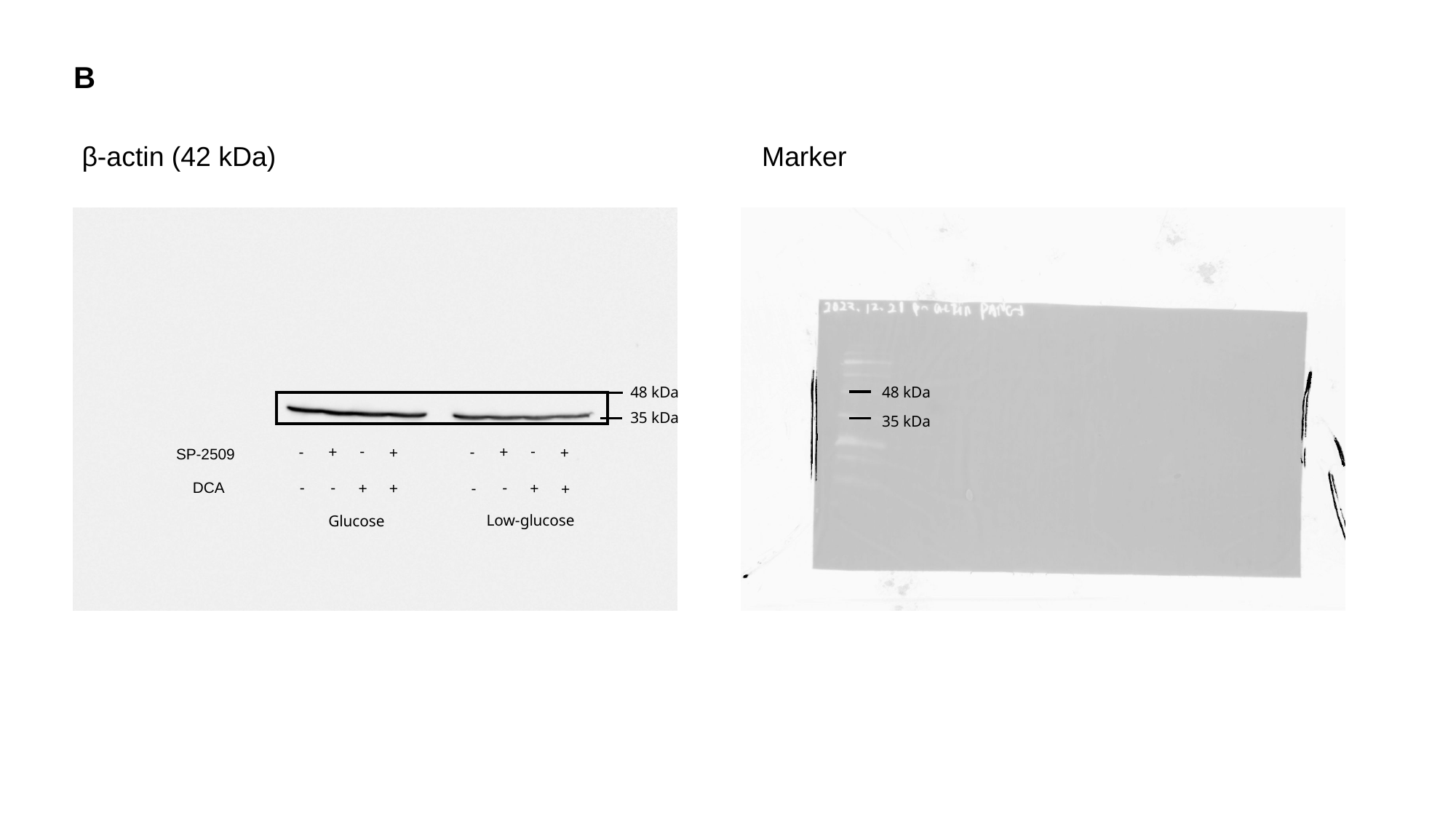

B
β-actin (42 kDa)
Marker
48 kDa
48 kDa
35 kDa
35 kDa
-
-
-
-
+
+
+
+
SP-2509
-
-
DCA
-
+
-
+
+
+
Low-glucose
Glucose

## Slide 6
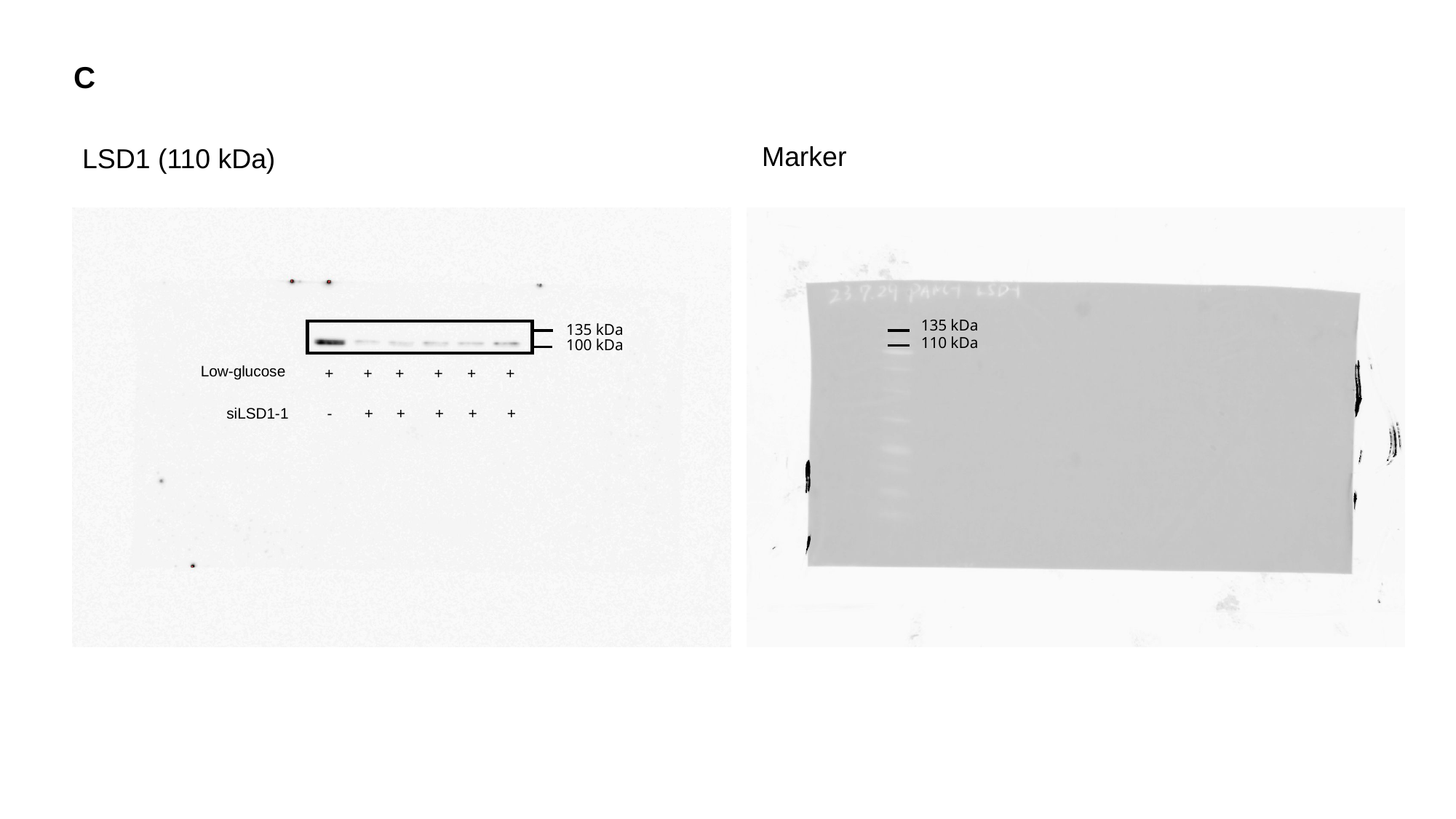

C
Marker
LSD1 (110 kDa)
135 kDa
135 kDa
110 kDa
100 kDa
Low-glucose
+
+
+
+
+
+
siLSD1-1
-
+
+
+
+
+

## Slide 7
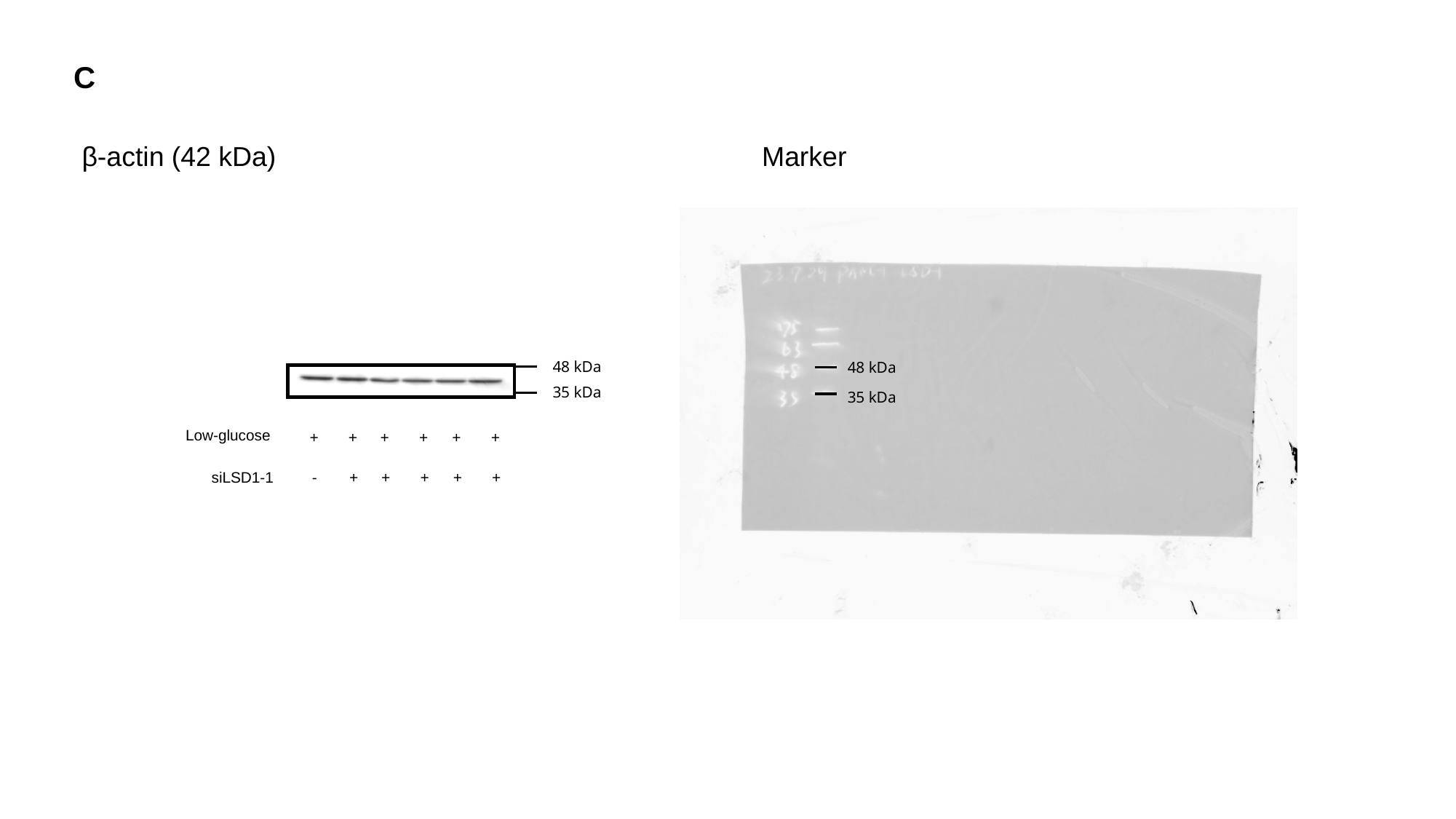

C
β-actin (42 kDa)
Marker
48 kDa
48 kDa
35 kDa
35 kDa
Low-glucose
+
+
+
+
+
+
siLSD1-1
-
+
+
+
+
+

## Slide 8
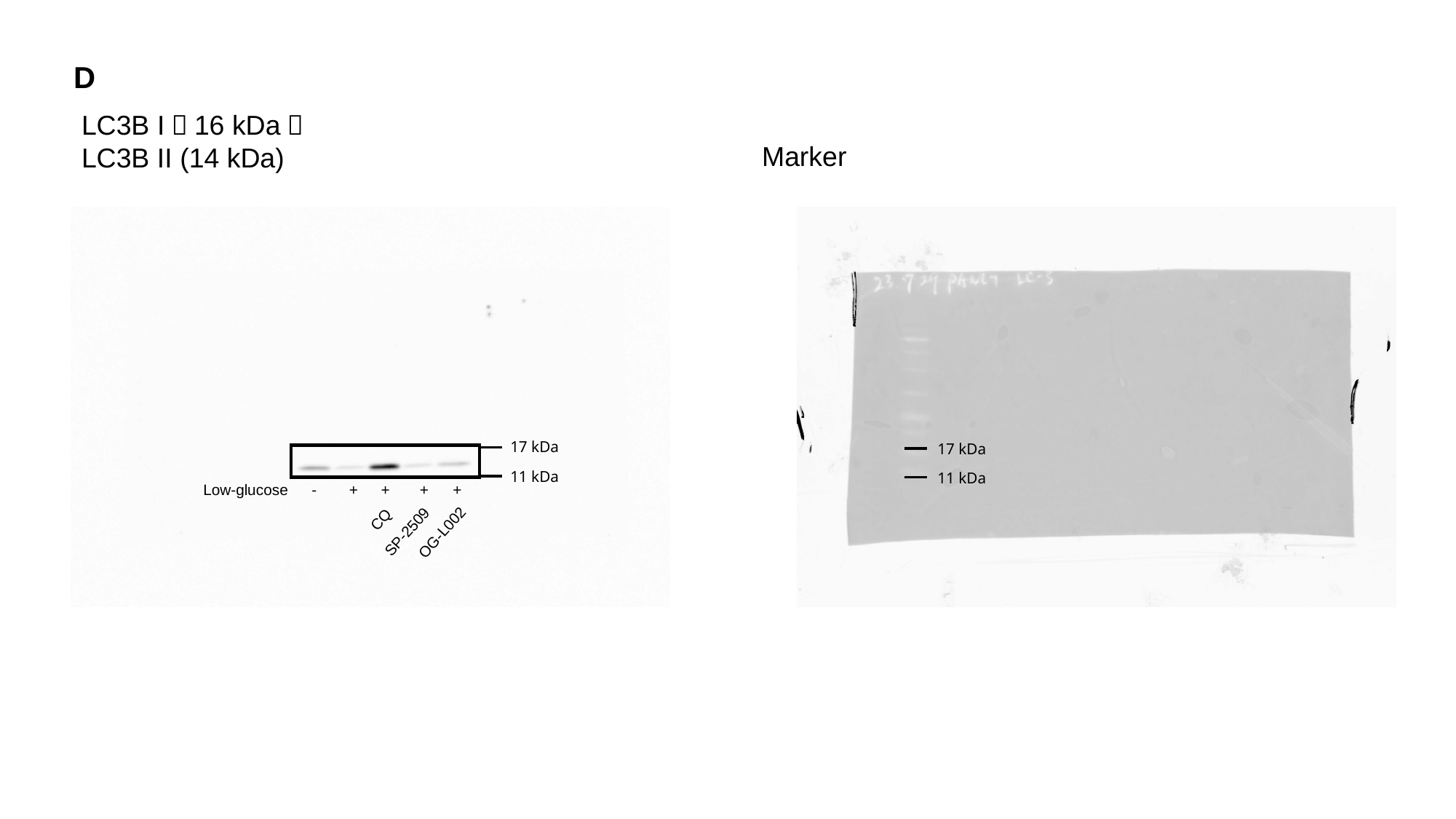

D
LC3B I（16 kDa）
LC3B II (14 kDa)
Marker
17 kDa
17 kDa
11 kDa
11 kDa
Low-glucose
-
+
+
+
+
CQ
SP-2509
OG-L002

## Slide 9
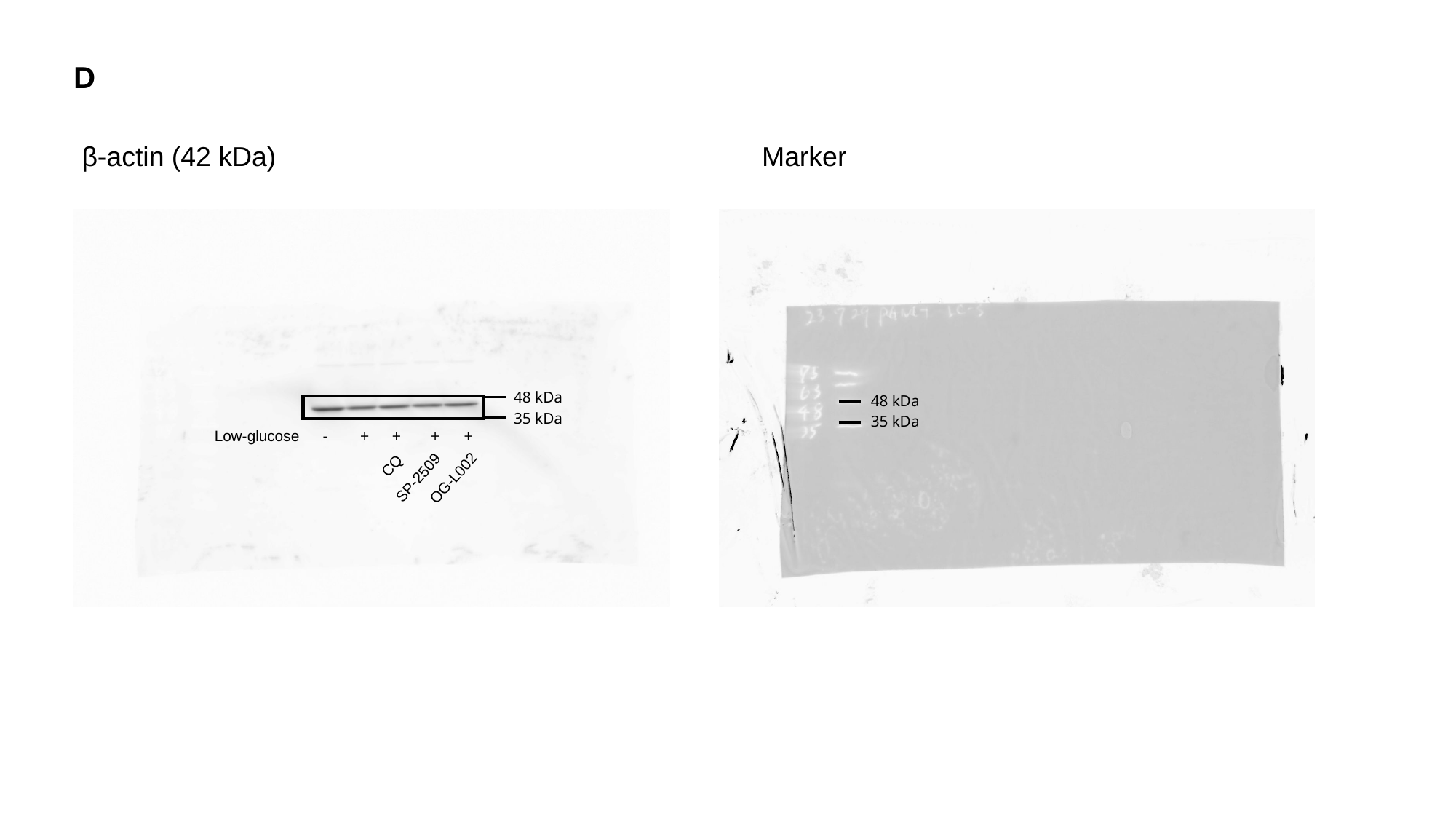

D
β-actin (42 kDa)
Marker
48 kDa
48 kDa
35 kDa
35 kDa
Low-glucose
-
+
+
+
+
CQ
SP-2509
OG-L002

## Slide 10
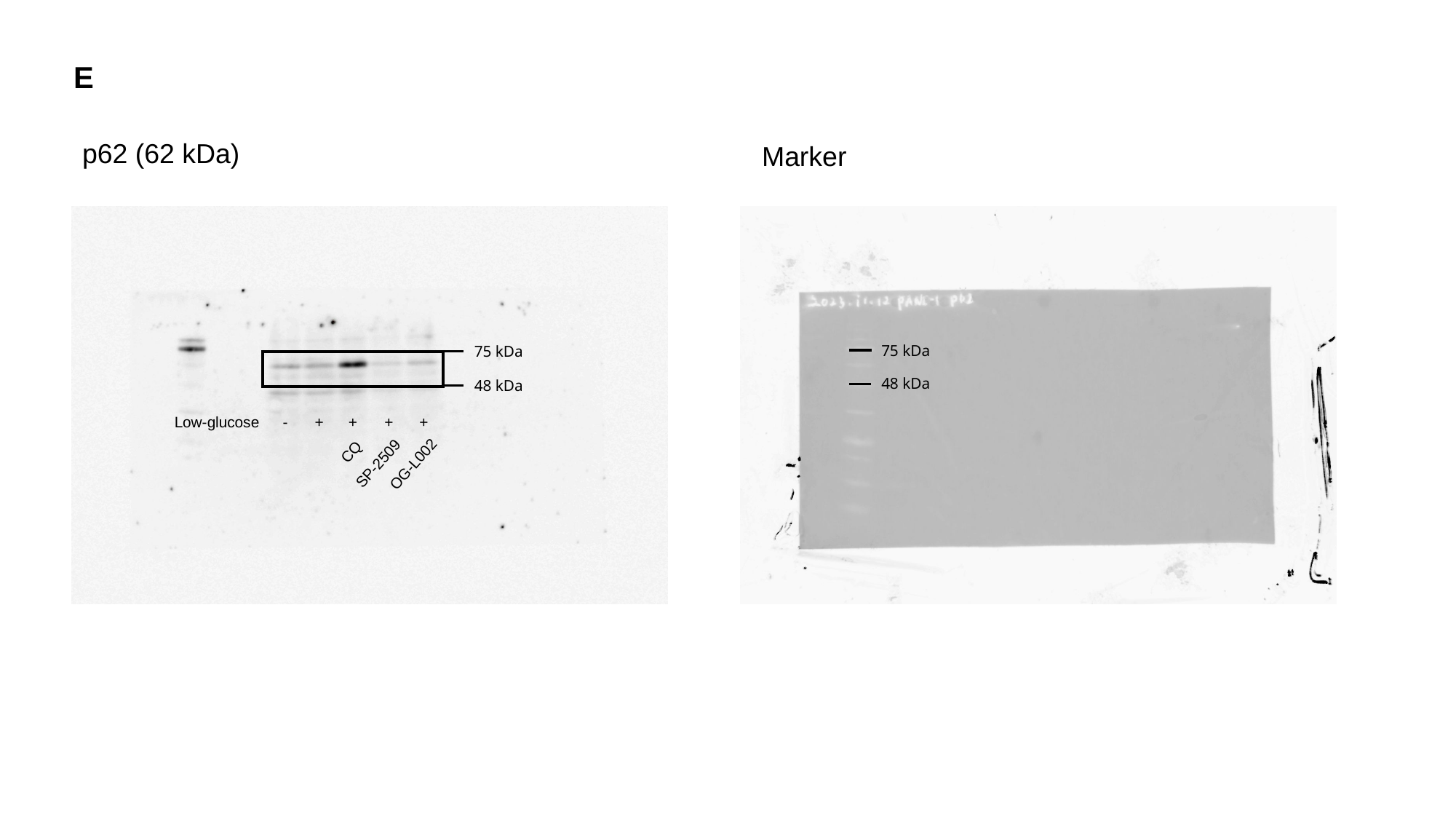

E
p62 (62 kDa)
Marker
75 kDa
75 kDa
48 kDa
48 kDa
Low-glucose
-
+
+
+
+
CQ
SP-2509
OG-L002

## Slide 11
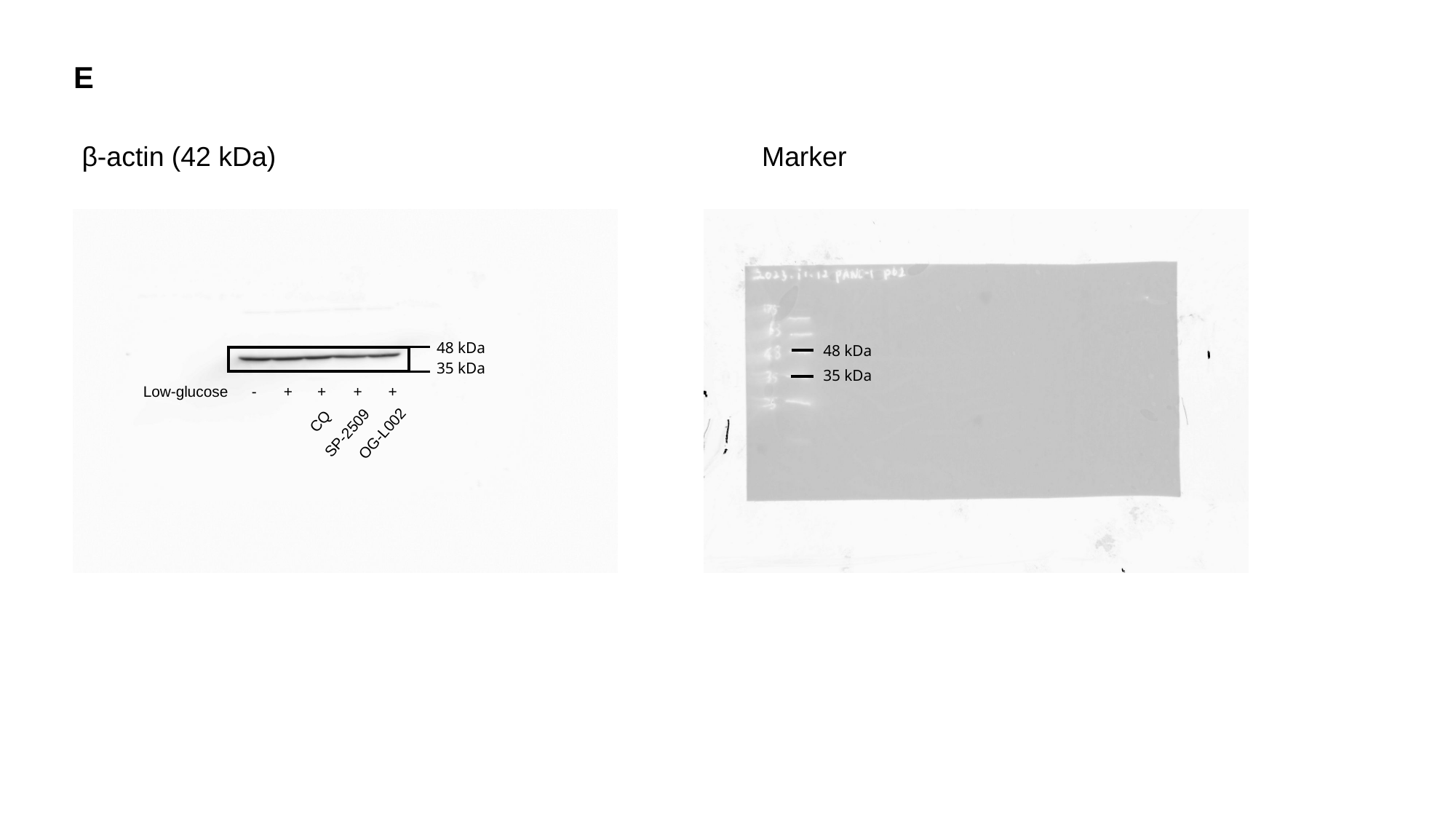

E
β-actin (42 kDa)
Marker
48 kDa
48 kDa
35 kDa
35 kDa
Low-glucose
-
+
+
+
+
CQ
SP-2509
OG-L002
